# Newly identified sex chromosomes in the *Sphagnum* (peat moss) genome alter carbon sequestration and ecosystem dynamics

**DOI:** 10.1038/s41477-022-01333-5

**Published:** 2023-02-06

**Authors:** Adam L. Healey, Bryan Piatkowski, John T. Lovell, Avinash Sreedasyam, Sarah B. Carey, Sujan Mamidi, Shengqiang Shu, Chris Plott, Jerry Jenkins, Travis Lawrence, Blanka Aguero, Alyssa A. Carrell, Marta Nieto-Lugilde, Jayson Talag, Aaron Duffy, Sara Jawdy, Kelsey R. Carter, Lori-Beth Boston, Teresa Jones, Juan Jaramillo-Chico, Alex Harkess, Kerrie Barry, Keykhosrow Keymanesh, Diane Bauer, Jane Grimwood, Lee Gunter, Jeremy Schmutz, David J. Weston, A. Jonathan Shaw

**Affiliations:** 1 Genome Sequencing Center, HudsonAlpha Institute for Biotechnology, Huntsville, AL USA; 2 Biosciences Division, Oak Ridge National Laboratory, Oak Ridge, TN USA; 3 Department of Energy Joint Genome Institute, Lawrence Berkeley National Laboratory, Berkeley, CA USA; 4 Department of Crop, Soil, and Environmental Sciences, Auburn University, Auburn, AL USA; 5 Department of Biology, Duke University, Durham, NC USA; 6 Arizona Genomics Institute, University of Arizona, Tucson, AZ USA; 7 Earth and Environmental Sciences Division, Los Alamos National Laboratory, Los Alamos, NM USA

**Keywords:** Abiotic, Comparative genomics, Phylogenetics

## Abstract

Peatlands are crucial sinks for atmospheric carbon but are critically threatened due to warming climates. *Sphagnum* (peat moss) species are keystone members of peatland communities where they actively engineer hyperacidic conditions, which improves their competitive advantage and accelerates ecosystem-level carbon sequestration. To dissect the molecular and physiological sources of this unique biology, we generated chromosome-scale genomes of two *Sphagnum* species: *S. divinum* and *S. angustifolium*. *Sphagnum* genomes show no gene colinearity with any other reference genome to date, demonstrating that *Sphagnum* represents an unsampled lineage of land plant evolution. The genomes also revealed an average recombination rate an order of magnitude higher than vascular land plants and short putative U/V sex chromosomes. These newly described sex chromosomes interact with autosomal loci that significantly impact growth across diverse pH conditions. This discovery demonstrates that the ability of *Sphagnum* to sequester carbon in acidic peat bogs is mediated by interactions between sex, autosomes and environment.

## Abstract (web summary)

This description of the *Sphagnum* (peat moss) genome includes a newly discovered sex chromosome. Interactions among sex, autosomes and environment show direct effects on *Sphagnum* growth which influences global carbon sequestration.

## Main

*Sphagnum* (peat moss) is both an individual genus and an entire ecosystem. *Sphagnum*-dominated peatlands are estimated to cover ~3–5% of the Northern Hemisphere boreal zone, yet store ~30% of the total global terrestrial carbon pool^1^. *Sphagnum* grows most abundantly in bogs and fens, where they engineer peatland habitats through acidification (via cation exchange for nutrient uptake) and depletion of oxygen to promote their own persistence and dominance^2^. Within bogs, *Sphagnum* species display niche preferences, growing at different heights above the water table (‘hummock’ mounds and ‘hollow’ valleys) and pH levels. This community microtopography is characteristic of *Sphagnum*-dominated peatlands where species habitat is phylogenetically conserved such that closely related species occupy similar niches^3,4^ which correlates with differences in growth, carbon sequestration and tissue decomposability. For these well-documented niche differences among species, *Sphagnum* and their bogs have long served as a model for studies of community assembly, stress physiology and carbon sequestration^5,6^; efforts which have recently been bolstered by ecological genomics and biogeochemical experimental innovations^7^.

In addition to species genetic differentiation, *Sphagnum* community assembly and within-species trait variation appear to be controlled in part by sex-ratio biases, where sexes are differentially adapted to local environments^8^. Sex, in haploid-dominant life-cycle bryophytes that have been examined, is determined by U/V (U, female; V, male) sex chromosomes that segregate 1:1 among spores during meiosis^9^. However, the mechanism for sex determination in *Sphagnum* has not yet been elucidated. While a balanced sex ratio is expected within any bryophyte habitat, skewed ratios are often observed (evidenced by either phenotypic or genotypic observations)^10^, particularly within stressful environments. These biases have important implications on effective population sizes and in extreme cases could result in population collapse^11,12^. Given that bryophyte sex ratios are influenced by extreme environments and *Sphagnum* engineers harsh, unfavourable conditions within bogs, *Sphagnum* comparative genomics provides a unique opportunity to investigate the underlying genetic components of bryophyte sex-determination and sex-ratio bias.

To facilitate genetic analysis of carbon sequestration and stress responses in peatlands and understand how *Sphagnum* responds to environmental stress (both native and self-generated), we developed the first chromosome-scale, de novo genome assemblies for *S. angustifolium* (subgenus *Cuspidata*) and *S. divinum* (subgenus *Sphagnum*). Genome sequencing and genetic map construction enabled the discovery of a minuscule (~5 megabase (Mb)) sex chromosome (chr. 20; V chromosome) that is one-quarter the size of other chromosomes, shares conserved gene order (synteny) with autosome chr. 7 and is derived from ancient whole-genome rearrangements. To study how *Sphagnum* contends with abiotic stress encountered in peat bogs, reference genotypes were exposed to laboratory-simulated pH stress, finding that species endemic to hummock and hollow niches differentially respond to alkalinity and acidity through hormone expression and plasmodesmata-mediated cell transport. Investigation of the effect of pH stress on *Sphagnum* physiology in our F_1_-haploid pedigree population found that quantitative trait loci (QTLs) that impacted growth were dependent on U/V chromosome inheritance, providing a direct link between peatland environmental conditions, carbon sequestration and sex-ratio biases that are commonly observed in bryophytes.

## Results

### *Sphagnum* represents an uncharacterized lineage of plants

Peat accumulation within bogs is primarily linked to growth, biomass deposition and low rates of decomposition. These traits, as well as niche and pH preferences among *Sphagnum* species are phylogenetically conserved. While tremendous variation exists across the five *Sphagnum* subgenera^13^ based on previously explored phylogenetic relationships and niche evolution^3,14^, for reference genome sequencing we selected two haploid genotypes, one from the ancestral hummock clade: subgenus *Sphagnum* (*S. divinum*; recently reclassified within the *S. magellanicum* species complex^15^) and the other from ancestral hollow clade: subgenus *Cuspidata* (*S. angustifolium*; previously *S. fallax*—misclassified at the time of collection; genotyped using marker data from ref. ^16^). Although *Sphagnum* diverged from other mosses millions of years ago (Ma), within the genus these references represent diverged lineages which diversified during the Miocene (7–20 Ma; ref. ^17^) and contain a large swath of *Sphagnum* functional ecological variation. Both were sequenced to ~70× consensus long read (CLR) PacBio coverage and assembled into highly contiguous chromosome sequences (Supplementary Table 1): the *S. divinum* and *S. angustifolium* genome assemblies were 439 Mb (contig N50: 17.5 Mb) and 395 Mb (contig N50: 17.4 Mb) in size, respectively. This is consistent with *k*-mer based genome size estimates for each reference of 424 and 367 Mb, respectively. Chromosomes were scaffolded for *S. angustifolium* from a high-density genetic map consisting of 2,990 genetic markers in 20 linkage groups (chromosomes 1–20). Gene content, order and orientation were then projected onto the *S. divinum* assembly to separately order contigs into 20 chromosomes. Each genome was also annotated with a combination of RNA-seq evidence-based and ab initio gene models, finding 25,227 primary gene models in *S. divinum* and 25,100 in *S. angustifolium*.

**Fig. 1:**
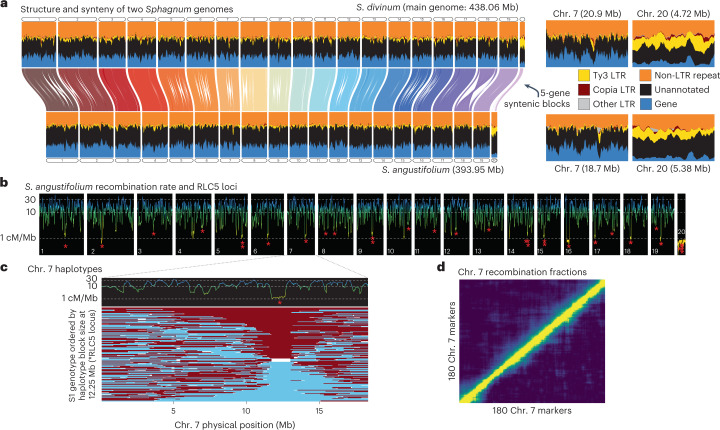
Comparative genomics of *Sphagnum*. **a**, Syntenic mapping between chromosomes, comparing gene density and repeat content. The orientation of chr. 9 (marked *) is reversed for visualization purposes. Chr. 7 and chr. 20 are duplicated with expanded axes to the right of the main plot to highlight the differences in repeat content. **b**, *S. angustifolium* recombination rate (calculated from the *S. angustifolium* genetic map) with putative centromere positions, denoted with red asterisks showing RLC5 cluster positions. Lines are coloured on the basis of *y* axis position to better highlight regions of low recombination (yellow) **c**, Zoomed in look at the RLC5 cluster region on chr. 7. Top panel shows recombination rate from the *S. angustifolium* genetic map (coloured by position on *y* axis), showing a drop in recombination coinciding with the RLC5 cluster. Bottom panel shows the recombination haplotypes (maroon and blue) within the F_1_-haploid pedigree (*n* = 184; denoted on the *y* axis), finding no recombined haplotypes in the region overlapping with the RLC5 cluster. **d**, Recombination/LOD score heatmap for chr. 7 to show high recombination rate in pedigree and tight linkage among markers. Source data

Comparison of chromosomes between *S. divinium* and *S. angustifolium* showed that, despite their divergence, high collinearity between genomes is maintained (Fig. 1a). Long contiguity of the genomes (contig N50: 17.5 and 12.1 Mb, respectively), paired with high synteny and annotation protein BUSCO scores (Viridiplantae: 98.3% each), show that the genome assemblies are high quality and the most contiguous non-vascular plant genomes produced thus far^18–21^. Interestingly, *Sphagnum* genome synteny does not extend to any other bryophyte or vascular plant lineages investigated (Supplementary Fig. 1), a result consistent with the findings of ref. ^19^ with *Anthoceros* hornwort genomes (*Sphagnum*–*Anthoceros* divergence: 496 Ma). This is in direct contrast, however, with the moss *Physcomitrium patens* and liverwort *Marchantia polymorpha* where gene colinearity can still be observed with other land plants^22,23^.

We observed typical bryophyte chromosome structure^22–25^ in these genomes. Repeat-rich pericentromeres (typical of angiosperms) were conspicuously absent while gene density was largely uniform across the genome, ranging between 20% and 25% (Fig. 1a). Genome-wide repeat content (~30%), with unclassified and terminal inverted repeat CACTA superfamily being the most abundant types (7.8% and 6.8%, respectively; Fig. 1a), was also similar to existing bryophyte genomes. Further, bryophytes have been shown to lack typical centromeric structures that are usually located via large arrays of tandem duplicated genes and increases in repeat density. In the *P. patens* genome, the authors noted unique *Copia*-like elements that clustered in distinct locations within each chromosome. These clusters were primarily composed of full-length and truncated RLC5 long terminal repeat (LTR) elements in tight clusters on each chromosome^22^. The same feature was described in green algae (*Coccomyxa subellipsoidea*), which was believed to be a centromeric structure^26^. To determine whether RLC5 clusters were present in *Sphagnum*, the RLC5 *Copia* sequence was extracted from the *Ceratodon purpureus*^18^ genome and was used to mask each *Sphagnum* genome. Each chromosome in both *S. angustifolium* and *S. divinum* possessed at least one dense RLC5 cluster locus, with some chromosomes having additional satellite clusters as well (Fig. 1b and Supplementary Table 2). A genome-wide scan of recombination across the *S. angustifolium* genome shows that the RLC5 clusters generally coincide with reduced recombination (Fig. 1b). For example, close inspection of the RLC5 loci on chr. 7 found no recombination, with two separate non-recombining haplotypes present (Fig. 1c). Given that each chromosome possesses a RLC5-dominated and non-recombining repeat cluster suggests that these LTR *Copia* elements function as a highly conserved centromere structure, whose evolution can be traced back to green algal ancestors.

A sequenced F_1_-haploid pedigree (derived from a single, field-collected sporophyte) of 184 *S. angustifolium* genotypes revealed a highly dense genetic map structure and high recombination rate in *Sphagnum*. The *S. angustifolium* genetic map is populated with 2,990 genetic markers and a total length of 5,396 centimorgan (cM) (Fig. 1b,d). The average recombination rate of the genome is 10–30 cM per Mb, or an average physical distance of 73 kilobases (kb) per cM, which is an order of magnitude higher than most vascular plants^27^ and appears to be a feature of mosses: the *P. patens* genetic map and recombination rate is similar to *Sphagnum* (5,432 cM; 27 linkage groups; ~11 cM per Mb)^22^, while the genetic map of *M. polymorpha*, a liverwort, contains roughly one-third of the recombination (76–111 cM per ~20 Mb chromosome)^23^. Given *Sphagnum’s* propensity for hybridization^28^, increased recombination may accelerate adaptation to environmental stresses and facilitate purging of deleterious alleles carried through linkage drag^29^.

### *Sphagnum* genetic diversity and phylogenetics

**Fig. 2:**
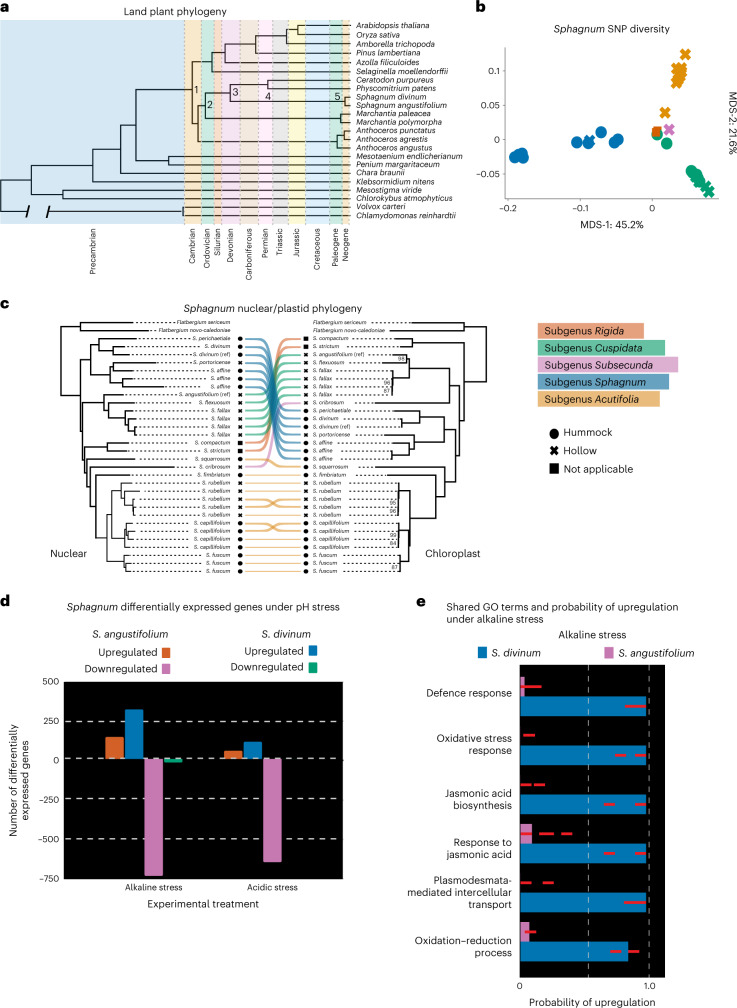
*Sphagnum* phylogenetics and response to pH stress. **a**, Fossil calibrated land plant phylogeny, with the branch separating the chlorophyte algae *Chlamydomonas* and *Volvox* from other species shortened for clarity and showing only terminal tips representative of major vascular plant lineages. Node ages (Ma) of note include: (1) Bryophyte divergence (515 Ma), (2) liverwort–moss divergence (473 Ma), (3) Sphagnopsida divergence (391 Ma), (4) *P.* *patens*–*C. purpureus* divergence (268 Ma) and (5*) Sphagnum* radiation (16 Ma). **b**, *Sphagnum* diversity panel SNP MDS plot. Species are coloured by subgenera and niche ecosystem preference (closed circle, hummock; cross, hollow). **c**, Phylogenetic relationships among haploid samples in the diversity panel using nuclear and chloroplast data suggest cytonuclear discordance. Branch support reflects ultrafast bootstrap values and nodes not labelled received maximal support. **d**, The pH stress response among *S. angustifolium* and *S. divinum*. **e**, Sign test among shared GO terms under alkaline stress. Results show that genes with shared terms are upregulated in *S. divinum* and downregulated in *S. angustifolium*. Red dashed lines represent the 95% confidence intervals. Source data

**Extended Data Fig. 1:**
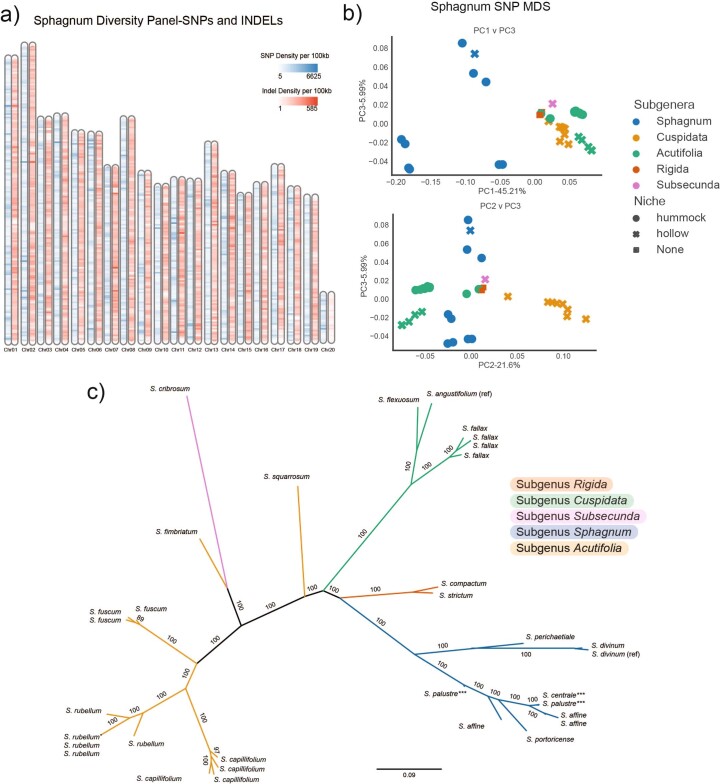
*Sphagnum* diversity panel genomic variation. **a**) *Sphagnum* diversity panel chromosome variation. SNPs and INDELS are relative to the *S. angustifolium* reference genome. The minor allele frequency (MAF) < 0.05 and sites with > 20% missing data were excluded. Any variant within 25 basepairs of a repeat element was excluded. Figure shows variant counts within non-overlapping 100 Kb windows. **b**) Multidimensional scaling (MDS) plots for *Sphagnum* SNP variation, relative to the *S. angustifolium* reference genome. Principal coordinates (PCs) were calculated after subsetting SNPs based on linkage disequilibrium (LD-see methods for description). Each point are colored by subgenera and shaped by environmental niche preference. **c**) Unrooted maximum likelihood SNP phylogeny for the *Sphagnum* diversity panel. Branch color represents the subgenus, branch support values represent the ultrafast bootstrap values, and branch lengths represent the number of substitutions per site. Polyploid species are labeled with asterisks (***). Source data

**Extended Data Fig. 2:**
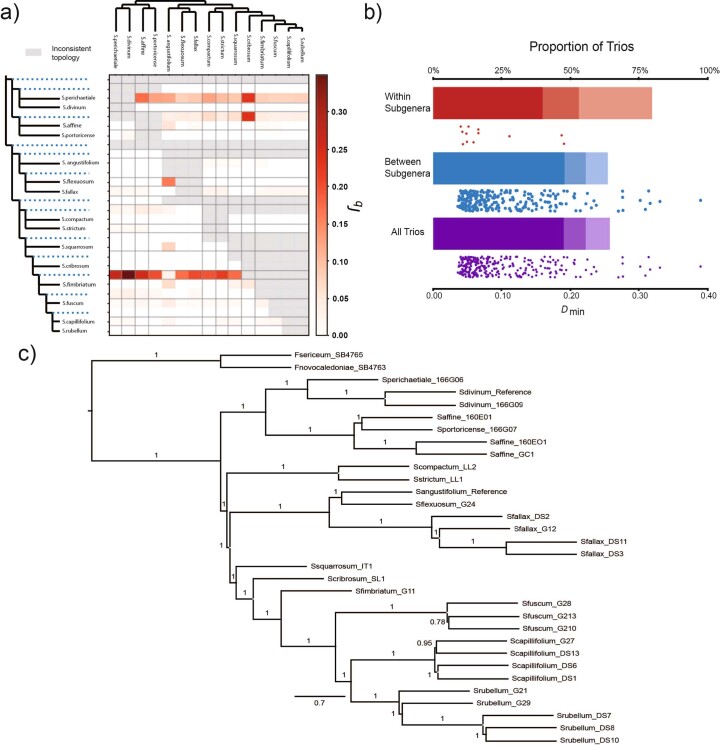
*Sphagnum* phylogenetics and introgression. **a**) Plot of *f*-branch statistics (*f*_b_) showing excess allele sharing between branches of the *Sphagnum* phylogeny (y-axis) and extant species of *Sphagnum* (x-axis). Dotted lines on the y-axis represent the most recent common ancestor for branches underneath each line. The maximum likelihood tree generated from 16,171 orthologs was used to calculate the f-branch statistic. Matrix entries colored by *f*-branch values are significantly different (*P*<0.05) than zero. Tests that are inconsistent with the given tree topology are shaded in grey. **b**) *D*_min_ statistics for trios of haploid *Sphagnum* species (same subgenus, different subgenera, all species) estimated from SNP data. Histograms represent the proportion of trios for which *D*_min_ is significantly different from zero (*P*<0.05; <0.01; <0.001) based on shading (lightest to darkest, respectively). Dots represent *D*_min_ values for significant trios. **c**) Phylogenetic relationships of *Sphagnum* resequencing samples (sample ID appended to species name) estimated from maximum likelihood ortholog genealogies using ASTRAL. Branch support values reflect local posterior probability and branch lengths are in coalescent units. Source data

Bryophytes, being one of the earliest plant clades^30^ to colonize land ~500 Ma, have undergone morphological and genetic diversification to contend with stresses associated with terrestrial life. To explore these evolutionary relationships, we constructed a land plant phylogeny among orthologues using IQ-TREE 2 (ref. ^31^; Fig. 2a and Supplementary Fig. 2). Divergence time estimation using fossil calibrated rates suggests that the two *Sphagnum* species represented by our references diverged ~16 Ma, which coincides with Miocene era cooling in North America that possibly led to *Sphagnum* diversification and radiation^17^. Reconstructing the evolutionary history within *Sphagnum* has remained a difficult task due to complex patterns of gene flow, incomplete lineage sorting and introgression^28^. To separate these phylogenetic signals, we sequenced a *Sphagnum* diversity panel (17 species in 35 accessions; Supplementary Table 3) representing each subgenera (*Acutifolia, Cuspidata, Rigida, Sphagnum* and *Subsecunda*). Alignment to *S. angustifolium* found 5,155,719 single nucleotide polymorphisms (SNPs) and 834,730 insertions/deletions (indels) across the panel, evenly distributed across chromosomes (Extended Data Fig. 1a). Visualization of the SNP variation using multidimensional scaling (MDS) shows the first two principal axes (45% and 22% of total variance explained) separate the largest taxonomic clades (*Acutifolia, Cuspidata* and *Sphagnum*) (Fig. 2b), with axes two and three (6% total explained variance) separating niche preference among subgenera (Extended Data Fig. 1b). Using 16,171 orthologues among *Sphagnum* species and non-*Sphagnum* peat mosses (*Flatbergium* spp.), the nuclear phylogeny presented strong conflict with the chloroplast phylogeny (Fig. 2c) suggesting evidence of past introgression (Extended Data Fig. 2a,b).

Ecosystem engineering is one of the primary mechanisms that *Sphagnum* uses to gain a competitive advantage over other organisms. One strategy used by *Sphagnum* to achieve this is acidification of their environment by cation exchange which keeps biomass inaccessible to microbial decomposition^3^. Given pH preference among *Sphagnum* subgenera, significant divergence among functional groups and thousands of genes we discovered bearing signature of positive selection among hummock and hollow lineages (hummock, 3,806 genes; hollow, 1,759 genes; Supplementary Table 4), we hypothesize that gene expression regulatory network evolution would both underlie pH preferences and respond to altered edaphic conditions. Using the land plant phylogeny, investigation of gene families found that 3,865 gene families (Supplementary Table 5) were expanded (average 3.6 *Sphagnum* genes per orthogroup versus 2.5 in land plants) in the most recent common ancestor of *Sphagnum*, with significant gene ontology (GO) enrichments for plant signal transduction (GO:0007165; adjusted *P* = 1.5 × 10^–3^) and response to stress (GO:0006950; adjusted *P* = 2.5 × 10^–2^). When exploring the effect of pH exposure (pH 3.5 and 9.0; Fig. 2d) on gene expression among *S. divinum* and *S. angustifolium*, pathways related to plant hormone signal transduction (plasmodesmata-mediated transport and jasmonic acid biosynthesis and response) were differentially expressed (Fig. 2e and Supplementary Tables 6 and 7), with genes associated with plasmodesmata-mediated transport being a top enriched target for transcription factors (TFs) in *S. divinum*. In mosses, both jasmonic acid (and its upstream precursor 12-oxo-phytodienoic acid; ref. ^32^) and plasmodesmata-mediated transport has been directly linked to phytohormone response to abiotic stress^33–35^, suggesting these phytohormone and cell-to-cell signalling pathways are highly conserved among vascular and non-vascular plants.

### Whole-genome duplications and the origin of a sex chromosome

**Fig. 3:**
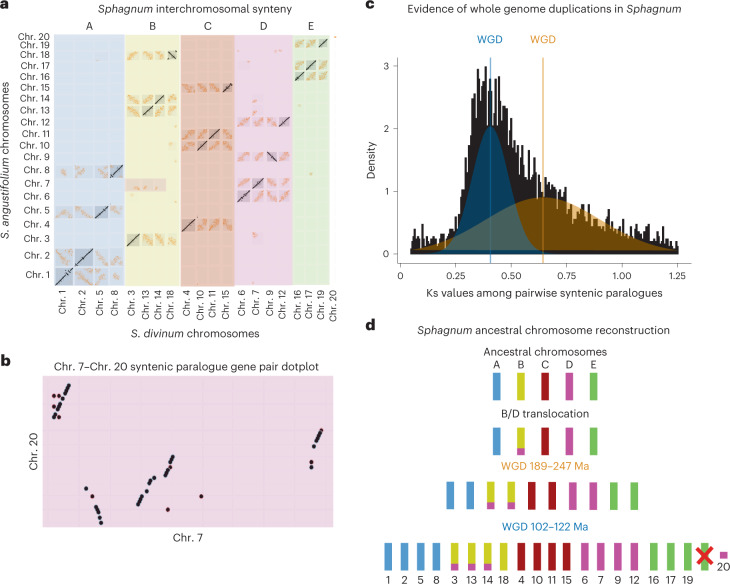
WGDs and ancestral chromosome reconstruction in *Sphagnum*. **a**, Interchromosomal synteny between *S. divinum* and *S. angustifolium*. *S. divinum* chromosomes are re-ordered to group paralogous chromosomes together while *S. angustifolium* chromosomes are arranged in increasing order (1–20). Ancestral B–D synteny on chr. 3, 13 and 14 is highlighted. **b**, Synonymous mutation rate among paralogous gene pairs in *S. divinum*. Two distributions derived from WGD are shown with the median of each peak (0.406; 0.643) marked with a coloured vertical line. **c**, Paralogous gene pairs among chr. 7 and chr. 20. Chr. 20 shares best-hit synteny with chr. 7. **d**, Ancestral chromosome reconstruction in *Sphagnum*. Little interchromosomal rearrangement has occurred after each WGD, except for the loss of one of the ancestral E chromosome homologues (noted with a red X). Genome duplication ages from ref. ^38^. Source data

Much like gene family expansion, whole-genome duplication (WGD) events provide the raw material for sub- and neo-functionalization and were important for terrestrial colonization from algae to land plants^36^. WGDs, while apparently pervasive in mosses, are difficult to detect due to their age^37^. *Sphagnum*, however, has highly conserved inter-/intragenomic synteny which enables ancestral chromosome reconstruction. Comparing *S. angustifolium* and *S. divinum* chromosome synteny reveals that, unlike *P. patens* with seven ancestral chromosomes^22^, *Sphagnum* possesses five ancestral chromosomes (A, B, C, D and E) that underwent two separate WGD events and a loss of a copy of ancestor E (4x ABCD; 3x E) to generate the modern-day *Sphagnum* genome. These ancestral chromosomes correspond to: A (chr. 1, 2, 5 and 8); B (chr. 3, 13, 14 and 18); C (chr. 4, 10, 11 and 15); D (chr. 6, 7, 9 and 12); and E (chr. 16, 17 and 19) (Fig. 3a). Additionally, chr. 7 maintains synteny with chr. 3, 13 and 14, resulting from a portion of ancestral chromosome D either being duplicated or translocated onto chromosome B before the first WGD, being maintained throughout each duplication, then lost from chr. 18. Chr. 20 (discussed below), being ~4× smaller (4.7 Mb) than chr. 1–19, shares best-hit synteny with chr. 7 (Fig. 3b) and is a possible relic from the ancestral B/D translocation/duplication and subsequent loss from chr. 18 (Fig. 3d).

To reconstruct the evolutionary history of each WGD, synonymous mutation rates (Ks) were calculated among syntenic paralogues among putative ancestral chromosomes. The most parsimonious number of Gaussian distributions among paralogues was two, coinciding with Ks peaks at 0.406 and 0.643 (Fig. 3c and Supplementary Table 8). This finding is consistent with the number of WGD events investigated by ref. ^38^, finding that *Sphagnum* and closely related peat moss genera *Flatbergium* and *Eosphagnum* shared two WGD events (189–247 Ma and 102–122 Ma; 95% CI), based on Ks values and reconstructed gene trees. After each WGD, the *Sphagnum* genome has remained remarkably stable, undergoing few large-scale chromosome rearrangements or translocations, with some chromosomes maintaining almost 1:1 chromosome-scale synteny with their duplicated counterparts (for example, chr. 6 and 7; Fig. 3a).

**Fig. 4:**
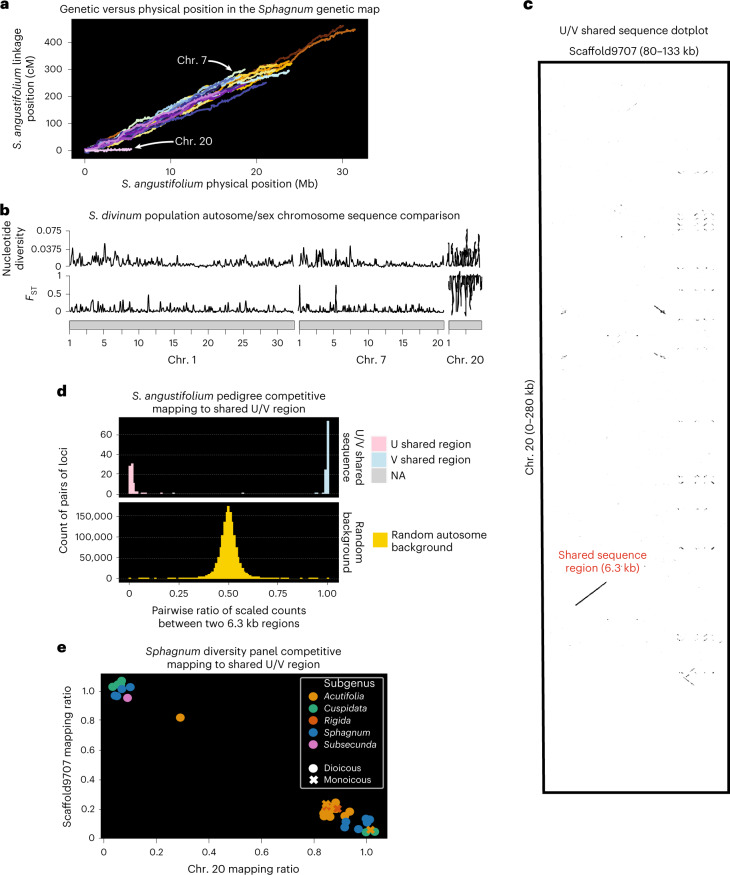
U/V chromosome detection and analysis. **a**, Recombination rate per chromosome, finding chr. 20 has a much lower rate of recombination than expected from the other 19 chromosomes. **b**, Sliding window analyses (100,000 bp window, 10,000 bp jump) of nucleotide diversity and *F*_ST_ between *S. divinum* chr. 20 SNP clusters. **c**, Exact *k*-mer dotplot with word size 15 for the shared sequence region between chr. 20 (putative V) and Scaffold9707 (putative U fragment), assembled from suspected female genotypes. **d**, *S. angustifolium* competitive mapping assay between chr. 20 and Scaffold9707. Ratio of reads mapped to the shared U/V region are shown, with individuals mapping to one sequence or the other (NA-ambiguous mapping ratio). Null distribution of autosome pairwise ratios is shown in yellow. **e**, *Sphagnum* diversity panel competitive mapping assay. Regardless of subgenera, individuals either mapped preferentially to the shared region of chr. 20 or Scaffold9707. Monoicous species (*S. squarrosum, S. compactum, S. strictum* and *S. fimbriatum*) each preferentially mapped to chr. 20. Positions on plot have been randomly ‘jittered’ by 0.1 units to improve readability among points. Source data

**Extended Data Fig. 3:**
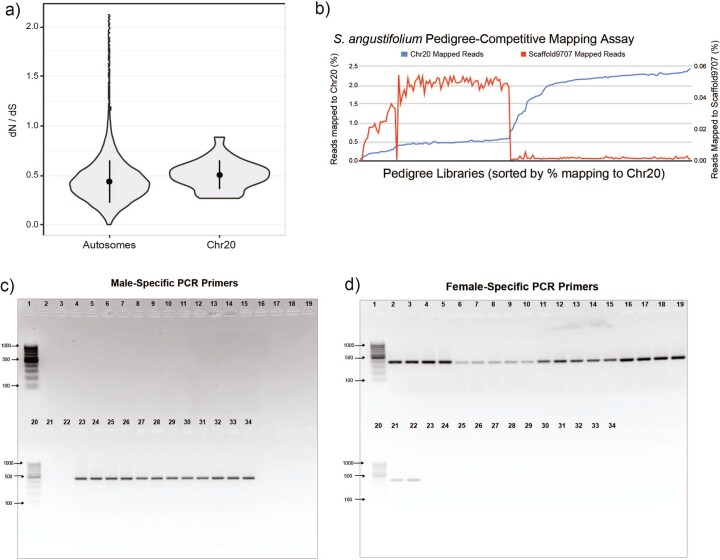
Analysis of Chr20 as a sex chromosome. **a**) Violin plot depicting gene-wide rate ratios of non-synonymous (dN) to synonymous (dS) substitution for genes on autosomes (n = 1,425) and chr.20 (n = 30) across the *Sphagnum* diversity panel. Higher values of dN/dS in genes on chr.20 suggest relaxation in the strength of purifying selection, positive selection, or a combination of both positive and relaxed purifying selection as compared to autosomal genes. Dots and bars represent mean values ± one standard deviation, respectively. **b**) *S. angustifolium* pedigree competitive mapping assay. Reads from each pedigree library were mapped to the genome assembly of *S.angustifolium*, along with the scaffold sequence Scaffold9707. Reads mapped to chr. 20 and Scaffold9707 are expressed as the total percentage per reads within the fastq file. **c** and **d**) Polymerase chain reaction results of male-specific (panel c) and female specific (panel d) primers used for amplification with *Sphagnum* samples of known sex (metadata details provided in Supplementary Table 12), Expected amplicon size for the PCR reaction is 444 bp (panel c) and 394 bp (panel d). PCR amplicons were separated on a 2% agarose gel, run for 2 hours at 80 volts. GeneRuler DNA ladder was run in lanes 1 and 20. DNA from female *Sphagnum* samples were loaded into lanes 2–19;21–22 and DNA from males was loaded into lanes 23–34. Individual gel results were not replicated but were independently consistent (for example male samples were 100% positive for male specific primers (panel c) and 100% negative for female-specific primers (panel d). Source data

In addition to 19 autosomal chromosomes, the assembly and genetic map of *S. angustifolium* first revealed the presence of another small chromosome (chr. 20), which was also present in *S. divinum* (chr. 20 5.4 Mb). Chr. 20 is approximately one-quarter the size of other chromosomes and displays suppressed recombination (2 cM; expected recombination was ~60 cM, based on size and recombination rate; Fig. 4a). Consistent with low recombination, chr. 20 also contains significantly more LTR content than chr. 1–19 (Ty3 16% versus 4%, Fisher’s exact test odds ratio 4.34, *P* < 0.001; *Copia* 1.2% versus 0.5%; Fisher’s exact test odds ratio 2.33, *P* < 0.001; Fig. 1a) and contains a low number of genes (coding sequence bases 8% versus 26%, Fisher’s exact test odds ratio 0.31; *P* < 0.001) that have non-synonymous (dN)/synonymous (dS) (dN/dS) ratios consistent with relaxed purifying selection (Wilcoxon rank sum test *P* = 0.011; Extended Data Fig. 3a).

One of the first systematic descriptions of chromosome structure within *Sphagnum* was conducted in 1955, where chromosome squashes typically described 19 bivalents and usually two minor (or M chromosomes) that were notably smaller^39^. *Sphagnum*, like most (60%)^40^ mosses, are dioicous (separate male and female haploid gametophytes) where sex is determined by U/V chromosome inheritance^41^. While tempting to assign chr. 20 to a sex chromosome on the basis of its characteristics (non-recombining, highly repetitive, relaxed purifying selection) and similarity to other bryophyte sex chromosomes^20,21,42^, the same genomic features are true for B chromosomes, which are pervasive throughout the plant kingdom^43,44^. As B chromosomes are cytogenetically inherited, we expected that the population genetic structure of polymorphism on a B chromosome would mirror variation found on the primary 19 chromosomes. Alternatively, moss U/V sex chromosomes that evolved in the ancestor of *Sphagnum* should possess high nucleotide diversity and strong patterns of divergence between females and males regardless of neutral genetic population structure of polymorphism on the autosomes. To test whether chr. 20 is a sex or B chromosome, we sequenced ten wild *S. divinum* samples collected across North America (Supplementary Table 9). SNPs on chr. 20 formed two distinct and highly diverged (*F*_ST_ > 0.95) clusters that did not match chr. 1–19 structures (Supplementary Fig. 3). This, in addition to high nucleotide diversity on chr. 20 between clusters (*π* = 0.0015; Fig. 4b and Supplementary Table 10), suggests that chr. 20 is a sex chromosome.

To definitively determine whether chr. 20 was either a U or V sex chromosome, we investigated its structure within the *S. angustifolium* F_1_-haploid pedigree, which contained the maternal parent of the cross. Mapping reads from the pedigree to chr. 20 showed a bimodal distribution (designated ‘low mapping’ and ‘high-mapping’; Extended Data Fig. 3b). As the maternal parent was a ‘low mapping’ individual, we suspected that the *S. angustifolium* reference is male and chr. 20 was a putative V chromosome. To investigate its U chromosome counterpart, reads from 20 individuals within the low-mapping chr. 20 distribution (including the maternal parent) were combined and assembled together (increasing coverage) using HipMer^45^. Protein sequences from chr. 20 were aligned to the HipMer scaffolds and any sequence that corresponded to each protein’s top alignment were extracted (coverage >60%). Scaffolds were then added to the *S. angustifolium* genome assembly for a competitive mapping assay among the pedigree population. One HipMer scaffold, Scaffold9707 (putative U chromosome segment—133,694 base pairs (bp)), displayed a similar, yet opposite, bimodal mapping pattern to chr. 20 (Extended Data Fig. 3b). Scaffold9707 is primarily composed of repeat content, except for 6.3 kb which contains a shared gene with chr. 20 (Sphfalx20G000800; calcium-binding EF-hand protein; 92% sequence identity) (Fig. 4c). Pairwise read count ratios (Supplementary Table 11) within this shared region found that reads from almost all individuals in the pedigree definitively map to one sequence or the other (except two, labelled NA), which is not significantly different than the expected 50:50 sex ratio of an F_1_-haploid population (female, 83; male, 91; exact binomial test *P* = 0.59). Pairwise count ratios between randomly sampled 6.3 kb regions across chr. 1–19 show no mapping bias (with the median shared U/V mapping ratio observed in 0.0006 autosome combinations) (Fig. 4d).

To conclusively test whether chr. 20 is the male V chromosome and Scaffold9707 represents a fragment of the female U chromosome, PCR primers were designed from the shared 6.3 kb region (Fig. 4c), intended to amplify a sex-specific ~400 bp target amplicon. DNA from vouchered *Sphagnum* samples (*n* = 28) where sex was known (through identification of sexual structures (females *n* = 16; males *n* = 12; Supplementary Table 12)) was extracted and used for PCR amplification. PCR results (Extended Data Fig. 3c,d) show that 100% of males and females generated their expected amplicon with no cross-reactivity, confirming that chr. 20 represents male (V) and Scaffold9707 represents female (U) sequences. While *Sphagnum* is predominantly dioicous, some species are monoicous^46^. To better understand sex-determination in monoicous *Sphagnum*, species within the diversity panel (which contains both dioicous and monoicous species) were competitively mapped against the shared region of chr. 20 and Scaffold9707 (Supplementary Table 13). Read mapping preference found two distinct groupings which were independent of phylogenetic relationship (Fig. 4e). Consistent with other bryophytes, the evolution of sex is not strictly related to changes in ploidy^47^. Sex could be confidently assigned in dioicous species (Supplementary Tables 3 and 13); however, all monoicous individuals tested mapped preferentially to chr. 20, suggesting that the potential role the V chromosome (or lack of U) may play a role in this transition. Lastly, to determine whether *Sphagnum* sex chromosomes share an origin with other U/V bryophytes, we built 36 gene trees from orthogroups that contained a gene annotated to chr. 20 in *Sphagnum* and a U- or V-linked gene in *Ceratodon*^18^ or *Marchantia*^21^. None of these showed a topology supporting a shared sex chromosome system but rather separate gene capture and loss events on the sex chromosomes in these species, suggesting sex chromosomes in *Sphagnum* may have arose independently (Supplementary Figs. 4 and 5).

### Sex-specific growth response to acidic bog conditions

**Fig. 5:**
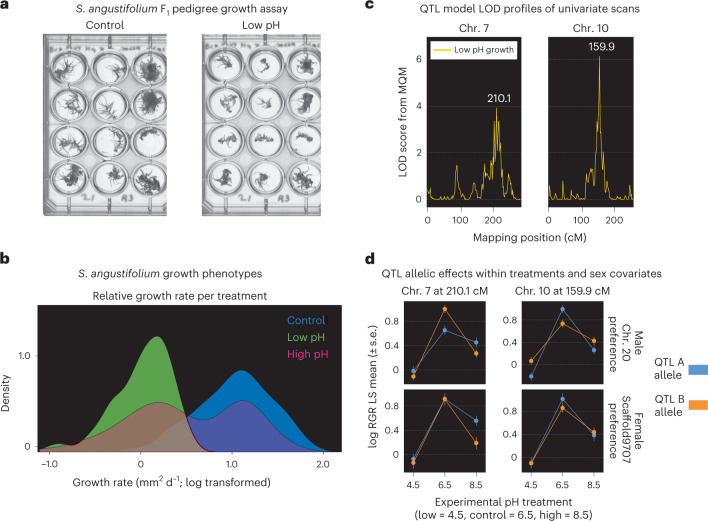
*S. angustifolium* pedigree QTL mapping in response to pH stress. **a**, Growth of pedigree genotypes under control and acidic stress conditions. **b**, Relative growth rates for the *S. angustifolium* pedigree under control, high (pH 8.5) and low (pH 4.5) pH conditions (*n* = 150). **c**, QTL mapping of low pH growth differences. Two QTL peaks were detected on chr. 7 and chr. 10. LOD scores, conditional on other QTL in a multiple QTL model, are presented. **d**, QTL effect plots. The connected line plots (shown with error bars) show the differences in growth for the variant alleles underlying each QTL loci. Each QTL is dependent on sex and autosomal parental allele (blue, A allele; orange, B allele). Panels are ordered by low (pH 4.5), control (pH 6.5) and high (pH 8.5) conditions, with data presented as mean values ± s.e. MQM, multiple QTL mapping; RGR LS, relative growth rate least squares. Source data

Despite its importance to global carbon cycling, the genetic mechanisms of the adaptation of *Sphagnum* to its engineered low pH environment is poorly understood. To infer the genetic loci that cause variation in the response of *Sphagnum* to pH stress, clones of the F_1_-haploid pedigree population were exposed to control (6.5 pH), acidic (4.5 pH) and alkaline (pH 8.5) conditions. We used relative growth rate (hereon ‘growth’, defined as occupied area within each imaging well (Fig. 5a) from time zero, log transformed) as the phenotype in each experimental treatment and calculated the relative response of each genotype as the difference between growth relative to the control (Supplementary Table 14). Growth was fastest under control pH (growth rate +0.88 mm^2^ d^−1^). Comparison among conditions found significant differences in growth (Kruskal–Wallis test; chi-square value 198.62; d.f. = 2, *P* < 0.001), which was slowest within the low pH treatment (growth rate −0.08 mm^2^ d^−1^; Student’s *t*-test, *t* = 21.4; *P* < 0.001). Within the high pH condition, there was a bimodal distribution of growth, where some individuals exhibited similar growth patterns to the control condition, while others grew similarly to those at low pH. Growth at high pH was significantly different from both the control (Wilcoxon rank sum test, *n* = 148, *W* = 15,595, *P* < 0.001) and low pH (Wilcoxon rank sum test, *n* = 148, *W* = 5,562, *P* < 0.001).

In addition to size and growth dimorphism^48,49^, bryophyte sex-ratio biases are often observed, where females tend to be favoured in a population^11,12,50^ (although male bias has been observed in *Sphagnum*)^8^. Given this previous research, we expected the presence of U or V genetic markers to be a strong predictor of growth under variable pH conditions. Unexpectedly, there were no significant differences in growth between sexes (Kruskal–Wallis test; chi-square value 0.80; d.f. = 1, *P* > 0.05), nor any significant effects of sex within any of the experimental treatments (all Wilcoxon rank sum tests, *P* > 0.05) (Fig. 5b). Our results also did not reveal differences in nucleotide diversity among inferred males and females within *S. divinum* wild populations (as predicted if mortality differences caused sex-ratio biases^11^; Supplementary Table 10) or sex-ratio bias within the pedigree (although this population was reared under artificial conditions).

Despite the lack of additive sex-biased phenotypic responses to pH conditions, it is possible that loci on the sex chromosomes or otherwise associated with cytoplasmic inheritance may interact with autosomal variation. Such sex (or cytoplasm)-by-autosome interactions are a common form of epistasis and may underlie genotype-by-environment interaction (G × E) to abiotic stresses in plants^51^. To test for epistatic interactions between autosome and sex chromosomes that cause differential growth responses, we conducted QTL mapping on the change in growth between experimental treatments and the control condition. In contrast to the lack of global sex-driven G × E, QTL scans conditioning on the additive effect of sex and testing for autosome–sex interactions detected two significant QTL peaks on chr. 7 (logarithm of the odds ratio (LOD) 3.8) and chr. 10 (LOD 6.4; Fig. 5c) for response to low pH stress.

While exposure to pH stress (both high and low) reduced growth across the pedigree, the effect of that stress was dependent upon sex and epistatic interactions at each major QTL loci. We found a significant interaction between sex-genotype and environment at QTLs on chr. 10 (*t* = 2.462, d.f. = 131.55, *P* < 0.05) and chr. 7 (*t* = 2.095, d.f. = 131.75, *P* < 0.05) when comparing the differences in growth between control and low pH conditions. Investigating these significant interactions in each sex separately found a significant G × E interaction among males at both loci (chr. 10 model test of fit *F*(1,155) = 23.885, *P* < 0.001; chr. 7 model test of fit *F*(1,155) = 13.21, *P* < 0.001). In contrast, growth in females was significantly impacted upon exposure to low pH but lacked any additive or epistatic effects at either loci (chr. 10 *F*(2,110) = 14.71, *P* < 0.001; chr. 7: *F*(2,110) = 14.71, *P* < 0.001). Considering each QTL peak was driven by sex-specific G × E interactions (often caused by trans-regulatory evolution), we investigated chr. 20 TFs with autosomal trans-effects. There were two annotated TFs on chr. 20, a mini-zinc finger (Sphfalx20G006700) and a Trihelix family protein (Sphfalx20G007700). Mini-zinc finger TFs have been broadly implicated in plant growth, root and flower growth and development, plant life span, fertility, and causes hormone insensitivity^52^, while trihelix TFs have been linked to biotic/abiotic stress response and tissue development^53,54^. The expression of both TFs was highly correlated (*r* > 0.8) with protein kinases within the QTL peak on chr. 10 (Supplementary Table 15). The rank change differences observed in growth among males (where an allele is beneficial in one environment but detrimental in another; Fig. 5d) is indicative of antagonistic pleiotropy^55,56^. This suggests separate adaptive strategies (specialist versus generalist) may be used by males and females in *Sphagnum* under abiotic stress conditions and could provide an explanation for why females (who lacked antagonistic pleiotropy) may be generally favoured in bryophyte populations.

## Discussion

The deep divergence between *Sphagnum* and other mosses and land plants in general is underscored by their genomics, biology and ecological function. Their ability to hybridize and generate unique allelic combinations through high recombination, paired with the ecosystem engineering allows *Sphagnum* to dominate across multiple biomes around the world. The key to the importance of *Sphagnum* for global carbon cycling is ecological differences between hummock and hollow species, which are directly impacted by differences in growth, cell wall structure and pH preference. The ability of *Sphagnum* to contend with both native and induced environmental stresses encountered within peatlands is directly linked to differential stress response, jasmonic acid precursors and cell-to-cell signalling via plasmodesmata, pathways that arose when plants first colonized land ~500 Ma (ref. ^24^).

An unexplored aspect of peatland carbon cycling is the effect of sex on growth and carbon sequestration in *Sphagnum*. In *C. purpureus*, females tend to produce thicker and larger leaves relative to males, which enables greater carbon sequestration (measured through leaf photochemistry)^49^. Bryophyte populations tend to skew toward one sex over the other^10,57^; however, hypotheses put forth to explain sex-ratio biases in bryophytes (for example, ‘shy’ males) have not accounted for epistatic interactions between U/V sex chromosomes and autosomes that result in differential response to environmental stresses. Local adaptation and maintenance of diversity in *Sphagnum* could be driven by sex-specific G × E interactions, resulting in plastic responses to stress within peatland conditions. Differential response between sexes could certainly result in sex-ratio bias if one sex can respond more effectively to persistent abiotic stress. Exploration of these principles in the *Sphagnum* pedigree population revealed a complex interaction between sex, genotype and environment, which would have remained undiscovered without the discovery of small U/V sex chromosomes in *Sphagnum*. These interactions were governed by an antagonistic pleiotropy and will require further study to fully elucidate their putative effects on sex-ratio biases, growth and carbon sequestration in *Sphagnum* and bryophytes in general. *Sphagnum*, with its small haploid genome, ease of maintenance and phenotyping in large-scale experimental populations^58^ and minuscule sex chromosomes linked to ancient whole-genome rearrangement, serves as a tractable model organism for not only niche ecosystem preference and carbon cycling but also sex chromosome evolution.

## Methods

### Plant material collection

Reference genome materials (*S. angustifolium* and *S. divinum*) were collected from the Marcell Experimental Forest (SPRUCE S1-Bog) (47.506639, −93.455897) by D. Weston in July 2016 and are maintained at the Duke herbarium (Duke University, NC, USA). Voucher information for *Sphagnum* samples included in the analyses is provided in Supplementary Table 3. Unextracted portions of each specimen have been deposited in the Duke herbarium.

### DNA extraction and sequencing

Genomic DNA from references grown in axenic cultures (derived from a single, surface-sterilized (70% ethanol), gametophyte stem) was extracted using the protocol of ref. ^59^ with minor modifications (2% CTAB buffer with proteinase K, PVP-40, sodium metabisulfite and beta-mercaptoethanol). DNA purity was measured with Nanodrop, DNA concentration measured with Qubit HS kit and DNA size was validated by pulsed field gel electrophoresis. Illumina libraries for the references were prepared as tight insert fragment libraries, 400 bp; 2 μg of DNA was sheared to 400 bp using the Covaris LE220 and size selected using the Pippin (Sage Science). The fragments were treated with end-repair, A-tailing and ligation of Illumina compatible adaptors (IDT) using the Kapa-Illumina library creation kit (Kapa Biosystems). The prepared libraries were quantified using the Kapa Biosystem next-generation sequencing library quantitative PCR (qPCR kit) and run on a Roche LightCycler 480 real-time PCR instrument. The quantified libraries were then prepared for sequencing on the Illumina HiSeq sequencing platform using a TruSeq Rapid paired-end cluster kit, v.2, with the HiSeq2500 sequencer instrument to generate a clustered flowcell for sequencing. Sequencing was performed on the Illumina HiSeq2500 sequencer using HiSeq Rapid SBS sequencing kits, v.2, following a 2 × 250 indexed run recipe.

*Sphagnum* PacBio (20 kb) libraries (from the same genotypes listed above for Illumina) were prepared with BluePippin size selection; 3.4 μg of genomic DNA was sheared to 20 kb using Covaris g-TUBEs. The sheared DNA was treated with exonuclease to remove single-stranded ends and DNA damage repair mix followed by end-repair and ligation of blunt adaptors using SMRTbell Template Prep Kit 1.0 (Pacific Biosciences). The library was purified with AMPure PB beads and size selected with BluePippin (Sage Science) at >6 kb cutoff size. PacBio sequencing primer was then annealed to the SMRTbell template library and sequencing polymerase was bound to them using Sequel Binding Kit 2.0. The prepared SMRTbell template libraries were then sequenced on a Pacific Biosystems Sequel sequencer using v.3 sequencing primer, 1 M v.2 SMRT cells and v.2.0 sequencing chemistry with 1 × 600 sequencing movie run times. Each of the genomes was sequenced to ~75× raw haploid coverage. The long-reads were assembled using MECAT (v.1.2)^60^ and subsequently polished using long-reads using ARROW (v.2.2.2)^61^.

Diversity panel samples (collected from the wild; Supplementary Table 3) were prepared as Illumina regular fragment, 600 bp. Plate-based DNA library preparation for Illumina sequencing was performed on the PerkinElmer Sciclone NGS robotic liquid handling system using Kapa Biosystems library preparation kit. A total of 200 ng of sample DNA was sheared to 600 bp using a Covaris LE220 focused-ultrasonicator. The sheared DNA fragments were size selected by double-SPRI and then the selected fragments were end-repaired, A-tailed and ligated with Illumina compatible sequencing adaptors from IDT containing a unique molecular index barcode for each sample library. The prepared libraries were quantified using the KAPA Biosystems next-generation sequencing library qPCR kit and run on a Roche LightCycler 480 real-time PCR instrument. The quantified libraries were then prepared for sequencing on the Illumina HiSeq sequencing platform using a TruSeq paired-end cluster kit, v.4. Sequencing was performed on the Illumina HiSeq2000 sequencer (yielding ~80× coverage per library) using HiSeq TruSeq SBS sequencing kits, v.4, following a 2 × 150 indexed run recipe.

DNA extraction from *S. angustifolium* pedigree samples were prepared similarly and sequencing libraries were constructed using an Illumina TruSeq DNA PCR-free library kit using standard protocols. Libraries were sequenced on an Illumina X10 instrument using paired ends and a read length of 150 bp and a sequencing depth of ~15× coverage.

### RNA experimental treatments

*S. divinum* and *S. angustifolium* grown in sterile tissue culture were used in all treatments.

There were a total of eight treatments with four replicates for *S. divinum* and two replicates for *S. angustifolium*. Before the experiment, 2.0 cm plugs of axenic *Sphagnum* were plated on BCD agar media at pH 6.5 and grown for 2 months in ambient temperature (20 °C) and a 350 photosynthetically active radiation (PAR) 12 h light/dark cycle. At 8:00 on the morning of the treatments, *Sphagnum* tissue was transferred to Petri dishes with 15 ml of appropriate BCD liquid media and placed in a temperature-controlled growth cabinet. Excluding the dark treatment, all samples were kept under 350 PAR for the duration of the experiment. Morning treatment samples (*S. divinum* only) were harvested 10 min after the light turned on and all other samples were harvested at 12:00. After each experiment the material was blotted dry, placed in a 15 ml Eppendorf tube, flash frozen in liquid nitrogen and stored at −80 °C until RNA extractions were completed.

For the control treatment, *Sphagnum* tissue was placed in a 22.05 cm^2^ Petri dish containing BCD media 6.5 pH and incubated in a growth cabinet at 20 °C and ambient light 350 PAR. To test low pH gene expression, the sample was placed in a 22.05 cm^2^ Petri dish containing 6.5 pH BCD media at 8:00. Each hour, the pH was gradually decreased until the sample was transferred to 3.5 pH media at 11:00. The samples were harvested at 12:00. This treatment was repeated for the high pH experiment, except the sample was gradually brought from 6.5 to 9.0 pH. Temperature experiments were controlled in growth cabinets with tissue in 22.05 cm^2^ Petri dishes containing 6.5 pH BCD media. The high temperature treatment began at 20 °C and, over 3 h, temperature was gradually increased to 40 °C. The low temperature treatment began at 20 °C and, over 3 h, was gradually decreased to 6 °C. To test drought effect on gene expression, tissue was placed on dry plates (no BCD media) for the duration of the experiment. Dark effect on gene expression was tested by placing material in a BCD-filled Petri dish in complete darkness from 8:00 to 12:00. To evaluate gene expression that is present during immature growth stages, a sporophyte was collected from the mother of the *S. angustifolium* pedigree and germinated on solid Knop medium under axenic tissue culture conditions. After 10 d of growth, plantlets were predominantly within the thalloid protonemata with rhizoid stage and flash frozen in LN2 for RNA isolation.

### RNA library preparation and sequencing

Total RNA was extracted from 100 mg of tissue with CTAB lysis buffer and the Spectrum Plant Total RNA Kit. Illumina RNASeq w/PolyA Selection, Plates—Plate-based RNA sample prep was performed on the PerkinElmer Sciclone NGS robotic liquid handling system using Illumina TruSeq Stranded mRNA HT sample prep kit using poly(A) selection of messenger RNA following the protocol outlined by Illumina in their user guide (https://support.illumina.com/sequencing/sequencing_kits/truseq-stranded-mrna.html) and with the following conditions: total RNA starting material was 1 μg per sample and eight cycles of PCR were used for library amplification.

The prepared libraries were quantified using the KAPA Biosystem next-generation sequencing library qPCR kit and run on a Roche LightCycler 480 real-time PCR instrument. Sequencing of the flowcell was performed on the Illumina NovaSeq sequencer using NovaSeq Xp v.1 reagent kits, S4 flowcell, following a 2 × 150 indexed run recipe.

### Pedigree growth and phenotyping

A sporophyte-bearing *S. angustifolium* mother MNSA5 (species verified by J. Shaw, Duke University) was collected at the SPRUCE experimental site within the S1-Bog on the Marcell Experimental Forest^62^ on 15 July 2012. Gametophytes were shipped to Oak Ridge National Laboratory where only attached sporophytes were removed from the gametophyte and kept in separate microcentrifuge tubes. For culturing, a single sporangium from a single female gametophyte was transferred to a sterile 1.5 ml microcentrifuge tube in a laminar flow hood, washed in 10% bleach solution for 5 min with periodic mixing, followed by 3× wash with sterile type I water. The surface-sterilized capsule was crushed with a sterile pipette tip in 200 µl of sterile water, diluted to 1 ml with additional sterile water and 200 µl of the diluted suspension was further diluted to 1 ml and spread on a BCD/agar plate topped with a disc of sterile cellophane. Plates were incubated at 25 °C in continuous light (~150 PAR m^−2^ s^−1^) to test germination. After 2 weeks, thalloid gametophytes were visible and individual protonema were transferred to single cells of 24-well plates containing solid BCD^63^. Eventually, a single capitula from each growing gametophyte (*n* = 600), as well as the maternal gametophyte was transferred to solid BCD or Knop plates or magenta vessels for maximum growth and maintenance at 25 °C 16 h days.

Before collection of phenotypes 2.0 cm plugs of axenic *S. angustifolium* were plated on BCD agar media pH 6.5 and grown for 2 months in ambient temperature (20 °C) and a 350 PAR 12 h light/dark cycle. A single capitulum of axenic *S. angustifolium* was added to each well of a 12-well plate with 2 ml of BCD media (pH 4.5, 6.5 or 8.5). The plates were placed into growth chambers with a 12 h light/dark cycle. Black and white images were collected weekly and surface area was measured using the ImageJ software^64^. The change in surface area was determined as a proxy for growth.

### Map construction

The 184 individuals sequenced in the pedigree population were aligned to the *S. angustifolium* pedigree, used for SNP calling as outlined below. A total of 2,856,328 SNPs were called, with 2,590,426 remaining after removing samples with >70% missing data (*n* = 12) and SNPs with >2% missing data. The cleaned SNP matrix was phased using the maternal ILEE library to 1,113,729 SNPs. This phased dataset showed little segregation distortion, so the genotype matrix was further subsetted to remove those with high linkage disequilibrium (>99.9%) and markers displaying 35–65% representation across the pedigree (*n* = 19,317). The genotype matrix was reformed as a QTL object using the github R package qtltools (v.1.2.0)^65^ and pairwise recombination fractions (RFs) among markers were calculated. Markers retained in the QTL object had no RF < 0.01 with any other marker (*n* = 5,969). Linkage groups (chr. 1–20) were formed using pairwise RFs with a minimum RF of 0.23 and a maximum LOD score of three. Markers on each linkage group were ordered using a travelling salesman problem solver, which minimizes the number of crossover events^66^. Lastly, after removing markers with segregation distortion patterns and those with high leverage (causing map expansion), markers closer than 1 cM apart were removed (*n* = 2,990).

### Chromosome construction and assessment

Genetic map markers (containing linkage and correlated cM position; *n* = 1,081,918) were extracted from the genetic map and aligned back to the PacBio assembly for *S. angustifolium*. Misjoins in the contigs were characterized by abrupt changes in linkage groups. Misjoins (*n* = 10) were broken, re-ordered and re-oriented on the basis of the genetic map. *S. angustifolium* (v.0.5 annotation) gene models were aligned to the newly oriented chromosomes to assign each protein a relative position along each chromosome. Proteins (*n* = 26,939) were then aligned to *S. divinum* PacBio contigs to identify misjoins (*n* = 4) which were broken, ordered and oriented into 20 chromosomes.

### Genome size estimation

Genome size of two samples (*S. angustifolium*, Illumina library ZCGA; *S. divinum*, Illumina library AGHCS) was estimated using *k*-mer of size 21. The Illumina reads were quality trimmed (for adaptors, low-quality bases) using inhouse scripts. Jellyfish (v.2.3.0)^67^ was used to estimate *k*-mer abundance and frequency distribution. Genome length and genome characteristics are estimated using Genomescope (v.2.0)^68^.

### Annotation

Transcript assemblies were made from stranded paired-end Illumina RNA-seq reads, ~598 M pairs of 2 × 150 bp for *S. divinum* and ~1.7 bp of 2 × 125 bp for *S. angustifolium*, using PERTRAN, which conducts genome-guided transcriptome short-read assembly via GSNAP (v.2013-09-30)^69^. Subsequently, 117,772 transcript assemblies for *S. divinum* and 122,707 transcript assemblies for *S. angustifolium* were constructed using PASA (v.2.0.2)^70^ from RNA-seq transcript assemblies above with respective genome. Loci were determined by transcript assembly alignments and/or EXONERATE (v.2.4.0) alignments of proteins from *Arabidopsis thaliana*, *Glycine max*, *Oryza sativa* Kitaake, *Setaria viridis*, *Vitis vinifera*, *Amborella trichopoda*, *M. polymorpha* and *Chlamydomonas reinhardtii*, high-confidence cross-species *Sphagnum* prelim gene models (*S. angustifolium* for *S. divinum* or *S. divinum* for *S. angustifolium*) and Swiss-Prot proteomes to repeat-soft-masked respective genome using RepeatMasker (v.open-4.0.7)^71^ with up to 2 kb extension on both ends unless extending into another locus on the same strand. Repeat library consists of de novo repeats by RepeatModeler (v.open1.0.11)^72^ on respective genome and repeats in RepBase. Gene models were predicted by homology-based predictors, FGENESH+ (v.3.1.0)^73^, FGENESH_EST (similar to FGENESH+, EST as splice site and intron input instead of protein/translated open reading frame (ORF)), EXONERATE^74^, PASA assembly ORFs (inhouse homology constrained ORF finder) and from AUGUSTUS (v.3.1.0) via BRAKER1 (v.1.6)^75^. The best scored predictions for each locus are selected using multiple positive factors including EST and protein support and one negative factor: overlap with repeats. The selected gene predictions were improved by PASA. Improvement includes adding untranslated regions, splicing correction and adding alternative transcripts. PASA-improved gene model proteins were subject to protein homology analysis to above-mentioned proteomes to obtain Cscore and protein coverage. Cscore is a protein BLASTP (v.2.2.26) score ratio to mutual best hit BLASTP score and protein coverage is highest percentage of protein aligned to the best of homologues. PASA-improved transcripts were selected on the basis of Cscore, protein coverage, EST coverage and its CDS overlapping with repeats. The transcripts were selected if Cscore was ≥0.5 and protein coverage ≥0.5, or if they had EST coverage but CDS overlapping with repeats is <20%. For gene models whose CDS overlaps with repeats for >20%, Cscore must be at least 0.9 and homology coverage at least 70% to be selected. The selected gene models were subject to Pfam analysis and gene models whose protein is >30% in Pfam TE domains were removed as were weak gene models. Incomplete gene models, low homology supported without fully transcriptome supported gene models and short single exon (<300 bp CDS) without protein domain nor good expression gene models, were manually filtered out.

### Transcriptome analysis

Illumina paired-end RNA-seq 150 bp reads were quality trimmed (*Q* ≥ 25) and reads <50 bp after trimming were discarded. RNA-seq samples with high-quality sequences were aligned to *S. angustifolium* and *S. divinum* reference genomes using GSNAP (v.2013-09-30)^69^ and counts of reads uniquely mapping to annotated genes were obtained using HTSeq (v.0.11.2)^76^.

Normalized count data were obtained using the relative log expression (RLE) method in DESeq2 package (v.1.14.1)^77^. Genes with low expression were filtered out by requiring ≥2 RLE normalized counts in at least two samples for each gene. Differential gene expression analysis was performed using the DESeq2 with adjusted *P* < 0.05 using the Benjamini–Hochberg method and a log fold change >1 as the statistical cutoff for differentially expressed genes. Expression data for all included treatments are available in Supplementary Tables 6 and 7 and Supplementary Fig. 6. Additional sign test (probability of upregulation) comparisons between shared GO terms per experimental treatment are provided in Supplementary Fig. 7.

Weighted gene co-expression networks were constructed using WGCNA R package (v.1.49)^78^ with variance stabilizing transformation expression data obtained from vst method in DESeq2 (v.1.14.1). We followed standard WGCNA network construction procedures for this analysis. Briefly, pairwise Pearson correlations between each gene pair were weighted by raising them to power. To select proper soft-thresholding power, the network topology for a range of powers was evaluated and appropriate power was chosen that ensured an approximate scale-free topology of the resulting network. The pairwise weighted matrix was transformed into topological overlap measure (TOM) and the TOM-based dissimilarity measure (1 − TOM) was used for hierarchical clustering. Initial module assignments were determined by using a dynamic tree-cutting algorithm. Pearson correlations between each gene and each module eigengene, referred to as a gene’s module membership, were calculated and module eigengene distance threshold of 0.25 was used to merge highly similar modules. These co-expression modules were assessed to determine their association with module eigengenes expression patterns distinct to tissues or conditions to gain insight into the potential biological role of each module.

GO enrichment analysis was carried out using topGO (v.2.48.0), an R Bioconductor package^79^ with Fisher’s exact test; only GO terms with a *P* < 0.05 were considered significant. To identify redundant GO terms, semantic similarity among GO terms was measured using Wang’s method implemented in GOSemSim (v.2.22.0)^80^, KEGG pathway enrichment analysis was performed on the basis of hypergeometric distribution test and pathways with *P* < 0.05 were considered enriched.

### *Sphagnum* diversity panel SNPs and indels

The paired-end sequences (2 × 150 bp) of the 35 samples were aligned to the *S. angustifolium* reference genome using bwa-mem (v.0.7.12)^81^. The aligned bams were deduped (PCR duplicates marked) using picard (v.2.17.2–0) tools (https://broadinstitute.github.io/picard/). The alignment statistics of the bams were obtained using samtools (v.1.9)^82^. Variant calling was performed using samtools mpileup (v.1.9) and Varscan (v.2.4.3)^83^ using a minimum depth of 8. Merging and filtering of the VCF was performed using bcftools (v.1.9)^84^. MDS coordinates were obtained for a random set of 50,000 SNPs obtained using LD pruning (--indep-pairwise 50 50 0.5) in PLINK (v.1.9)^85^. Polyploid samples were determined using variant frequency graphs (Supplementary Fig. 8) within CDS sequences and a minimum depth of 30.

### GENESPACE comparative genomics

Syntenic orthologues and paralogues among *S. divinum* and *S.angustifolium* were inferred via GENESPACE (v.0.9.4)^86^ pipeline using default parameters. In brief, GENESPACE compares protein similarity scores into syntenic blocks using MCScanX^87^ and uses Orthofinder (v.2.5.4)^88^ to search for orthologues/paralogues within synteny constrained blocks. Orthologue information is projected between reference genomes (Fig. 1a). To search for conserved gene synteny among *Sphagnum* and other published bryophyte genomes (*C. purpureus*, *M. polymorpha*, *P. patens*, *H. curvifolium*, *E. seductrix* and *A. agrestis* (Bonn))^18–22^, GENESPACE was run using default parameters. No conserved gene order was found among *S. angustifolium* and any other bryophyte genome (raw syntenic hits before syntenic block construction shown in Supplementary Fig. 1, with *H. curvifolium–E. seductrix* and *S. angustifolium–S. divinum* shown as positive controls. Similarly, to reconstruct ancestral chromosomes and infer WGDs, protein sequences within *S. divinum* hierarchical orthogroups were extracted from Orthofinder and aligned using MAFFT (v.7.487)^89^. Alignments were converted from amino acids into CDS sequences using pal2nal (v.13)^90^. Pairwise synonymous mutation rates (Ks) among sequences were calculated using seqinr (v.4.2-16)^91^. Mclust (v.5.4.10)^92^ was used to estimate the number of normal distributions present (*k* = 2) within the dataset on the basis of combined Ks values, based on Bayesian information criteria. Gene pairs (*n* = 5,094) were assigned to peaks (Ks = 0.406; 0.643) on the basis of their posterior distribution using normalmixEM in mixtools (v.1.2.0)^93^.

### RLC5 cluster detection

Putative centromeres within the *S. angustifolium* genome were detected using the RLC5 sequence extracted from the *C. purpureus* genome sequence^18^. Locations on each chromosome were discovered by masking the *S. angustifolium* genome with 20 bp *k*-mers from the RLC5 locus. Windows of 5 kb with a step size of 200 bp were slid across each chromosome. RLC5 regions (putative centromeres) are defined as five consecutive windows where >5% of bases are masked (Supplementary Table 2).

### Land plant phylogeny

To place *Sphagnum* in the broader context of land plant evolution, we obtained protein-coding loci from 36 genomes to reconstruct phylogeny. We used the primary transcript from each locus for genomes obtained through Phytozome v.13 (https://phytozome-next.jgi.doe.gov/) (Supplementary Table 17) and the longest isoform from each locus for the other genomes. Orthogroups among all species were inferred using Broccoli (v.1.2)^94^ and used to generate a complete set of gene trees estimated under maximum likelihood from DIAMOND (v.0.9.35.136)^95^ alignments using FastTree (v.2.1.11)^96^.

To exclude paralogues and analyse only putatively single-copy orthologues, the Yang–Smith pipeline^97^ was used to refine orthogroups. Briefly, tree-based refinement was performed to (1) mask in-paralogues, isoforms and redundant sequences, (2) trim outlier tips that probably represent assembly artifacts and (3) cut long internal branches to separate paralogous gene copies. For each round of refinement, codon alignments for each orthogroup were generated using translatorX (v.1.1–2)^98^ and MAFFT (v.7.487). Alignments were then trimmed to 0.1 column occupancy using trimAl^99^ (v.1.2rev59) and maximum likelihood trees were obtained with FastTree using only the first two codon positions due to saturation. Spurious tips were removed from the resulting trees with TreeShrink (v.1.3.2)^100^. Tips belonging to the same sample were then masked using the Yang–Smith script ‘mask_tips_by_taxonID_genomes.py’. To determine a suitable internal branch length for separating paralogous gene copies, we inferred orthologues using the ‘monophyletic outgroups’ method of Yang–Smith and used an adaptive threshold determined by the average branch length separating the outgroup from ingroup in trees that had all outgroups and at least half of the ingroup taxa^101^. These adaptive thresholds were used to cut longer internal branches of the maximum likelihood trees to separate paralogues. The entire refinement process was repeated after separating paralogues, producing a set of 3,230 orthologues using the ‘monophyletic outgroups’ method of the Yang–Smith pipeline and requiring at least half of all taxa to be present.

Codon alignments for these orthologues were generated and trimmed to 0.7 column occupancy as described previously. We estimated the species tree using a concatenated alignment of first and second codon positions across orthologues in IQ-TREE 2 (v.2.1.3)^31^ and determined the best partitioning scheme and substitution model using ModelFinder^102^. Branch support was determined using the ultrafast bootstrap method with 1,000 replicates. We estimated divergence times using the maximum likelihood tree and 12 fossil calibrations from ref. ^103^ (Supplementary Table 18) with treePL (v.1.0)^104^. The optimal smoothing parameter was chosen using cross-validation.

To model gene family evolution, we used the original orthogroup delimitations from Broccoli and reconstructed ancestral states of gene family occupancy under Wagner parsimony (gain penalty 1.0) using the program Count (v.9.1106)^105^. We considered a gene family to be expanded (contracted) if the orthogroup occupancy was greater (less) in the most recent common ancestor of *Sphagnum* than in the most recent common ancestor of mosses. Enrichment analysis of GO ‘biological process’ terms was performed by creating a custom GO term database for *S. divinum* v.1.1 using AnnotationForge (v.1.34.1)^106^ and using the enrichGO function in clusterProfiler (v.4.0.5)^107^ to analyse the *S. divinum* loci associated with expanded (contracted) gene families. The *P* values form the enrichment tests were adjusted using the Benjamini–Hochberg procedure and a term was considered enriched if the adjusted *P* was <0.05.

### Nuclear and chloroplast phylogeny of *Sphagnum*

To reconstruct the evolutionary history of samples within *Sphagnum*, we performed phylogenetic analyses using protein-coding loci from the two reference genomes (*S. angustifolium* v.1.1 and *S. divinum* v.1.1), 28 haploid resequencing assemblies and the outgroup transcriptomes from *Flatbergium novo-caledoniae* and *F. sericeum*^38^. The *Sphagnum* resequencing libraries BPHAT, BPHAZ, BPHBZ and BPHBS were excluded from these analyses because contamination and/or low coverage prohibited de novo genome assembly. Protein-coding sequences within the *Sphagnum* diversity panel were predicted using the GeMoMa (v.1.6.4)^108^ homology-based prediction pipeline (default parameters) on the basis of a constrained search, where the best hit locations of each *Sphagnum* transcript were extracted from each assembly with a 500 bp buffer.

The nuclear phylogeny was generated from the predicted protein sequences and refined using the Yang–Smith pipeline as described above, except that the Yang–Smith script ‘mask_tips_by_taxonID_transcripts.py’ was used to mask tree tips belonging to the same sample. We used the ‘monophyletic outgroups’ method from the Yang–Smith pipeline to identify 16,171 orthologues, requiring at least half of the ingroup taxa to be present. These sequences were concatenated and the phylogeny was estimated using IQ-TREE 2 (v.2.1.3)^31^ with model selection and branch support evaluated as described previously. To account for the possible effects of incomplete lineage sorting on phylogenetic reconstruction, we used the quartet-based method of ASTRAL (v.5.7.1)^109^ to summarize the maximum likelihood orthologue genealogies in a coalescent framework (Extended Data Fig. 2c).

To estimate the organellar phylogeny for samples in our dataset, raw reads were used to perform de novo assembly of chloroplast genomes with NOVOPlasty (v.2.6.7)^110^. For each plastid genome, contigs were manually aligned to the published *S. palustre* plastid genome (GenBank KU726621) and to each other to identify the inverted repeat boundaries and generate a single incomplete chloroplast genome sequence (with missing data represented by strings of Ns) including the long single-copy region, one copy of the inverted repeat and the small single-copy region. Plastid sequences were aligned with MAFFT and the phylogeny was estimated using IQ-TREE 2 with model selection and branch support evaluated as described previously. Using both the nuclear and plastid maximum likelihood trees, a cophylogenetic plot was generated with the R package phytools (v.0.7-90)^111^ in the R statistical programming environment (v.4.1).

### SNP phylogeny of *Sphagnum* and introgression

In addition to gene-based phylogenetic analyses, we performed SNP-based phylogenetic analyses to reconstruct the evolutionary history of *Sphagnum* (Extended Data Fig. 1c). Reads from resequencing samples were aligned to the *S. divinum* v.1.1 reference genome as outlined in the *Sphagnum* diversity panel section. Each sample was split from the multisample VCF and filtered to remove heterozygous sites using BCFtools (v.1.13)^84^. Individual samples were then filtered to keep only sites with a minimum depth of 10 (minDP = 10) and minimum genotype quality of 30 (minGQ = 30) using VCFtools (v.0.1.17). To include outgroups samples, we aligned transcriptome reads from *F. novo-caledoniae* and *F. sericeum*^38^ to the *S. divinum* v.1.1 genome using the two-pass mode of STAR (v.2.7.9a)^112^. Before alignment of these transcriptome reads, we used Trimmomatic (v.0.39) to remove bases on the 3ʹ ends of reads in the FASTQ files with a quality score threshold of 25 and kept reads longer than 40 bp after trimming. Duplicates in the STAR alignments were marked and sorted using Picard (v.2.26.2) (http://broadinstitute.github.io/picard). The alignments were then reformatted using the SplitNCigarReads function in GATK (v.4.2.2.0)^113^, variants were called using VarScan (v.2.3.9)^83^ and the VCF file was filtered to keep homozygous sites with a minimum depth of 10 and minimum genotype quality of 30.

Individual VCFs were combined to produce one multisample VCF containing only haploid samples with the two outgroups and another multisample VCF containing all *Sphagnum* samples (including polyploids). Each combined dataset was filtered to keep only autosomal sites with at least 80% of the samples genotyped. Sites were further filtered for a minor allele frequency of at least 0.05 and pruned for linkage disequilibrium using PLINK (v.1.90b6.24)^85^ with a window size of 50 variants, a window shift of 10 variants after each pruning step and a variance inflation factor threshold of 2.

The dataset containing all *Sphagnum* samples was used to infer phylogeny using IQ-TREE 2 as previously described. The dataset containing only haploid samples was used to test for the presence of admixture due to the robust presence of cytonuclear discordance in other analyses. The program Dsuite (v.0.4 r38)^114^ was used to calculate *D*-statistics (ABBA-BABA) and *f*_4_-ratios across the genome. As the true phylogeny of *Sphagnum* is unknown, we report the *D*_min_ statistic^115^. For a given species trio, *D*_min_ is the lowest value for *D* across all possible tree topologies and represents a lower bound for the amount of introgression. A *D*_min_ score >0 means that the evolutionary relationships between species in a given trio cannot be represented by a strictly bifurcating tree due to excess allele sharing (Extended Data Fig. 2b). As introgression between ancestral lineages can lead to correlated values of *D* across extant lineages, we used the *f*-branch metric to determine whether interspecific gene flow detected using *D*-statistics could reflect past introgression. We used the maximum likelihood tree from the analysis of the ‘monophyletic outgroups’ orthologue dataset to quantify *f*-branch. Significance testing was performed using the block jackknife method with 100 blocks and the resulting *P* values were adjusted using the Benjamini–Hochberg procedure (Extended Data Fig. 2a).

### Signatures of selection

We sought to detect signatures of natural selection across the genus *Sphagnum* by comparing the rates of dN and dS substitution in protein-coding genes. The goal of this analysis was to identify genes subject to positive selection during the evolution of hummock and hollow lineages. We obtained orthologues using the Yang–Smith pipeline as described in the previous section on phylogenetic reconstruction within the genus *Sphagnum* but used the ‘rooted ingroups’ method (16,910 orthologues) requiring at least half of the ingroup taxa to be present. For each gene, an in-frame codon alignment and the corresponding maximum likelihood gene tree was estimated using IQ-TREE 2 as described previously. Stop codons and frameshifts within codon alignments were masked with ambiguous nucleotide characters using MACSE (v.2.05)^116^.

Branches of each phylogenetic tree were designated as ‘foreground’ or ‘background’ for these tests, where foreground branches were those that we were interested in testing for evidence of positive selection. We assigned habitat designations to all terminal branches and performed ancestral state reconstruction to label internal branches of each gene tree corresponding to their marginal likelihood of being either hummock or hollow. Ancestral state reconstruction was performed using the rerooting method of ref. ^117^ under an equal rates model of transition probabilities using the phytools and evobiR (v.1.1)^118^ packages in the R statistical programming environment. Two sets of analyses were conducted within *Sphagnum*: one in which hollow lineages were specified as foreground and another in which hummock lineages were the foreground.

We used the method BUSTED^119^, implemented in HyPhy (v.2.5.32)^120^, to test each gene for signatures of positive selection. BUSTED is a branch-site test that aims to detect evidence of gene-wide positive selection along foreground branches of a phylogeny. Sites in each phylogenetic partition (foreground or background) were assigned to one of three omega (*ω* or dN/dS) classes, *ω*_1_ ≤ *ω*_2_ ≤ 1 ≤ *ω*_3_ and the likelihood of this model was compared to one constrained by the absence of a *ω*_3_ class on foreground branches. The *P* values from the BUSTED analyses were adjusted using the Benjamini–Hochberg method and a test was declared significant at *P* < 0.05, indicating that at least one site on at least one foreground branch was positively selected (Supplementary Table 4).

We also sought to determine if genes on chr. 20 have evidence for relaxed purifying selection. We used BUSTED without specifying foreground lineages to quantify dN/dS and performed a Wilcoxon rank sum test to assess whether the mean ratio in genes on chr. 20 was different from those on autosomes. We considered tests for orthologues that had loci from both reference genomes present (Extended Data Fig. 3a). Higher values of dN/dS in genes on LG20 relative to those on autosomes could suggest relaxation in the strength of purifying selection, the presence of or increase in the strength of positive selection or a combination of these factors.

### Chr. 20 genomic diversity

To examine the patterns of genomic variation per chromosome within *S. divinum* populations, DNA from ten genotypes collected across North America (Supplementary Table 9) was extracted, as noted above in DNA extraction and library preparation sections. SNP variation and MDS coordinates from each library were collected after aligning reads to the *S. angustifolium* reference (as noted in the *Sphagnum* diversity panel section). Variation on autosomes (chr. 1–19) followed rough geographic distributions, whereas variation on chr. 20 split into two clusters, independent of location. To examine patterns of genetic variation between clusters, we performed sliding window analyses using PopGenome (v.2.7.5)^121^ in R v.4.0.3. We calculated nucleotide diversity within and between clusters, as well as *F*_ST_ between clusters, using a window size of 100,000 bp with a jump of 10,000 bp. Windows were plotted using karyoploteR (v.1.16.0)^122^.

### Sex chromosome PCR confirmation

To find conserved regions of the genome for primer design, the transcript sequence of the gametologue (Sphfalx20G000800) was aligned to the genome assemblies across the diversity panel using BLAT (v.30)^123^ with default parameters. Diversity panel samples were binned together on the basis of suspected males and males (using their mapping ratio results (Supplementary Table 13)). The bounds of the top match alignment were extracted used bedtools (v.2.29.0)^124^ getfasta and combined for multiple sequence alignment using MAFFT (v.7.487)^89^. Gaps in the alignment were removed using Trimal (v.1.4.rev15; parameters: -gappyout)^99^. The conserved aligned regions among suspected male/female bins were used to design female (forward CCCTAGCTTCCAGCCAATTA, reverse CCTTCTTCTTGGCCTCATCTAC; expected amplicon size 394 bp) and male (forward TCCACAGAGGTGGACATAGA, reverse GTGGGATGAGAACTGGGATAAG; expected amplicon size 444 bp) primer sets for PCR. To determine whether the PCR primers were sex specific, *Sphagnum* samples (*n* = 28) where sexual structures were observed in the field (and confirmed under microscope (for example, antheridia and capsules)) were used for DNA isolation and PCR amplification. DNA was extracted from a single capitulum of each sample using the modified CTAB extraction process described in ref. ^125^. For each primer pair, genomic DNA was amplified by PCR in 30 μl volumes using KAPA HiFi HotStart ReadyMix and contained 50 ng of template DNA, a 0.25 mM concentration of each primer pair, 1 KAPA HiFi HotStart ReadyMix and molecular grade water. PCR amplifications were performed with the conditions 95 °C for 3 min, 25 cycles of 95 °C for 30 s, 58 °C for 30 s and 72 °C for 30 s and a final extension of 72 °C for 5 min. The PCR products were run in a 2% agarose gel at 80 V for 2 h (Extended Data Fig. 3c,d). GeneRuler (100 bp) size fragment DNA ladder (Thermo Scientific; SM0241) is included in gel lanes 1 and 20. Gel lane details per sample are found in Supplementary Table 12.

### U/V sex chromosome comparative genomics

To examine whether the *Sphagnum* sex chromosomes share an origin with other U/V species, we built gene trees to examine the topology. We used peptides for 57 mosses and liverworts from existing de novo assemblies from ref. ^18^ and genome annotations for *C. purpureus* v.1.1 (ref. ^18^), *P. paten*s v.3.3 (ref. ^22^), *M. polymorpha* v.6.1 (ref. ^21^) and as outgroups we used *Azolla filiculoides* v.1.1 (ref. ^126^), *Salvinia cucullata* v.1.2 (ref. ^126^) and *Selaginella moellendorffii* v.1.0 (ref. ^127^). We used OrthoFinder v.2.5.2 (ref. ^88^) in ultrasensitive mode to identify orthogroups that contained genes on chr. 20 in our *Sphagnum* genomes and were sex-linked in *C. purpureus* or *M. polymorpha*. We aligned each gene using MAFFT (v.7.471)^89^ with the parameter maxiterate set to 1,000 and using genafpair. We built gene trees using RAxML (v.8.2.12)^128^ with 100 bootstrap replicates and the model PROTGAMMAWAG. We visually assessed each tree to determine if the topologies supported that any *Sphagnum* chr. 20 genes were found in a monophyletic clade with other V-linked genes.

### QTL mapping

Quantitative loci mapping was performed in R/qtl (v.1.50)^129^ using the Haley–Knott regression method on hidden Markov model-calculated genotype probabilities. One-way and multiple QTL model scans were conducted on log-transformed phenotypes to correct for right-skewed distributions. In all QTL scans, sex was treated as a covariate as determined both by markers extracted from the genetic map for chr. 20, as well as the ratio of reads mapped to the shared region among chr. 20 and Scaffold9707 (as described in main text). Any sample where the marker data or mapping ratio were ambiguous was assigned ‘NA’. To determine the significance thresholds for each QTL, 1,000 permutations were performed. To estimate the effects of predicted sex (male and female), genotype at each QTL locus (A or B) and treatment (control and low pH) on log-transformed growth, we fit a univariate mixed linear model with all two- and three-way interactions, with a random effect of individuals to account for the same individual measured in two conditions. Sex-specific linear models were run with interactions, with goodness of fit compared between each. *S. angustifolium* genes within significance intervals are listed in Supplementary Table 15.

### Reporting summary

Further information on research design is available in the Nature Portfolio Reporting Summary linked to this article.

## Supplementary information

Supplementary Information Supplementary Figs. 1–8.

Reporting Summary

Supplementary Tables Supplementary Tables 1–18.

## Data Availability

Additional work to support the findings of this paper can be found in the Supplementary Figures and Tables. Sequencing libraries (Illumina DNA/RNA and PacBio CLR) are publicly available within the SRA. Individual accession numbers are provided in Supplementary Table 16, with additional data submitted under BioProject PRJNA799298. Genome assemblies and annotations (v.1.1) are freely available at Phytozome (https://phytozome-next.jgi.doe.gov/). These whole-genome shotgun projects have been deposited at DDBJ/ENA/GenBank under the accessions JAJQJK000000000 (*S. angustifolium*) and JAKJHR000000000 (*S. divinum*). The versions described in this paper are versions JAJQJK010000000 and JAKJHR010000000. Raw data used for analysis in this paper are freely available on figshare (10.6084/m9.figshare.21232100)^130^. Source data are provided with this paper.

## Source data

Source Data Fig. 1 Genome plot source data.
Source Data Fig. 2 Phylogeny, SNP and differentially expressed gene data.
Source Data Fig. 3 Raw blast and synonymous data.
Source Data Fig. 4 Fst and plot data.
Source Data Fig. 5 Genotype and phenotype QTL data.
Source Data Extended Data Fig. 1 Genomic and phylogenetic data.
Source Data Extended Data Fig. 2 Phylogeny and introgression data.
Source Data Extended Data Fig. 3 Chr. 20 analysis data.
Source Data Extended Data Fig. 3 Unprocessed PCR gels for panels c and d.

## Code availability

Scripts used for analysis in this paper are freely available on figshare (10.6084/m9.figshare.21232100)^130^.
