# Supplementary material for: Newly identified sex chromosomes in the Sphagnum (peat moss) genome alter carbon sequestration and ecosystem dynamics
Source: Nat Plants. 2023 Feb 6;9(2):238–54. doi: 10.1038/s41477-022-01333-5 (PMC9946827; doi:10.1038/s41477-022-01333-5)
Supplement: Supplementary file 1 — Supplementary Figs. 1–8. [file 41477_2022_1333_MOESM1_ESM.pdf]

nature plants

Article

https://doi.org/10.1038/s41477-022-01333-5

Newly identified sex chromosomes in the *Sphagnum* (peat moss) genome alter carbon sequestration and ecosystem dynamics

In the format provided by the authors and unedited

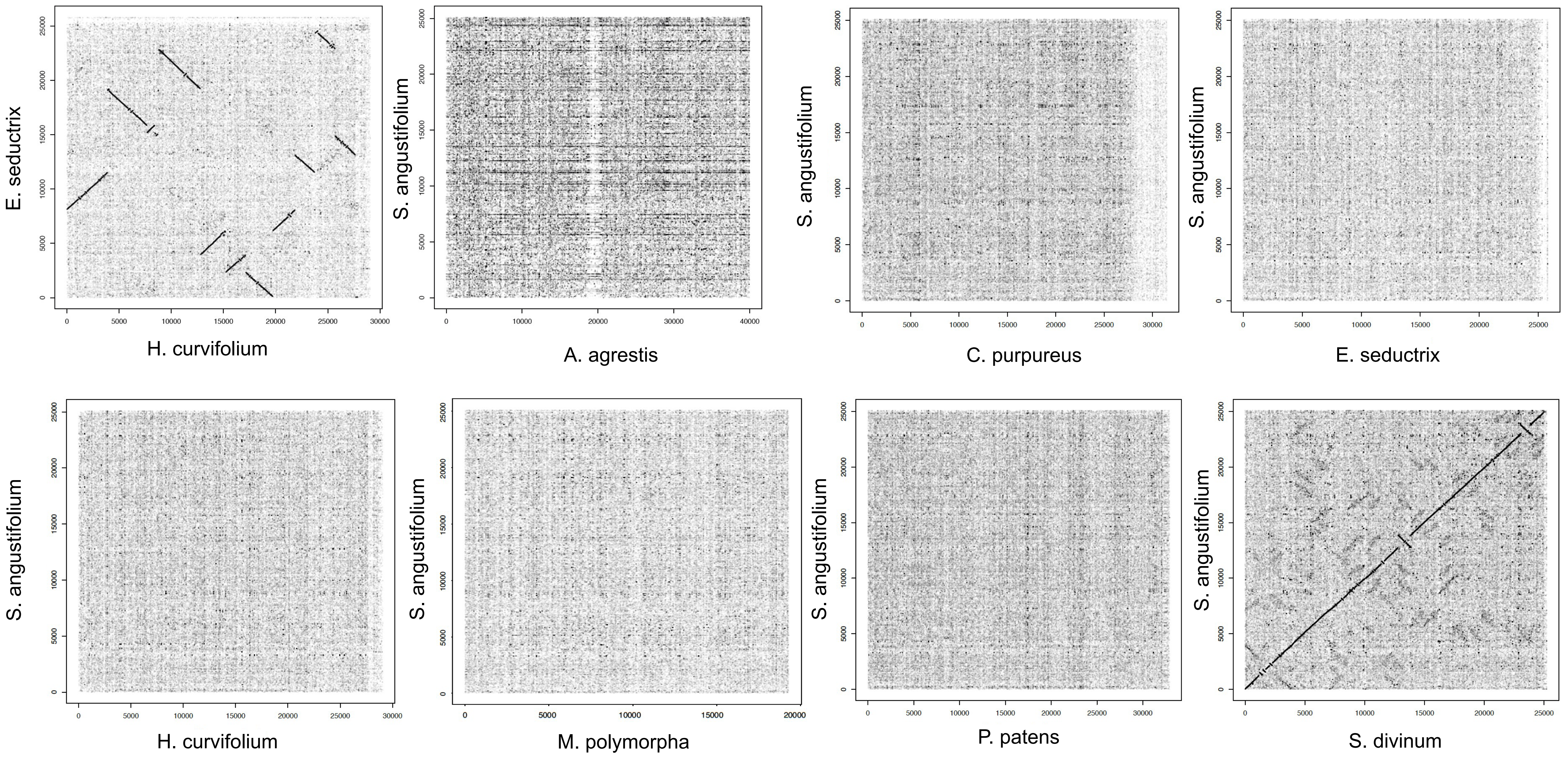

Supplemental Figure 1- Raw syntenic hits among peptides from GENESPACE among genomes: S. angustifolium, S. divinum, C. purpureus, M polymorpha, P. patens, H. curvifolium, E. seductrix, and A. agrestis (bonn). Order of peptide sequences on the X and Y axes is carried over from their annotation gff3 file.

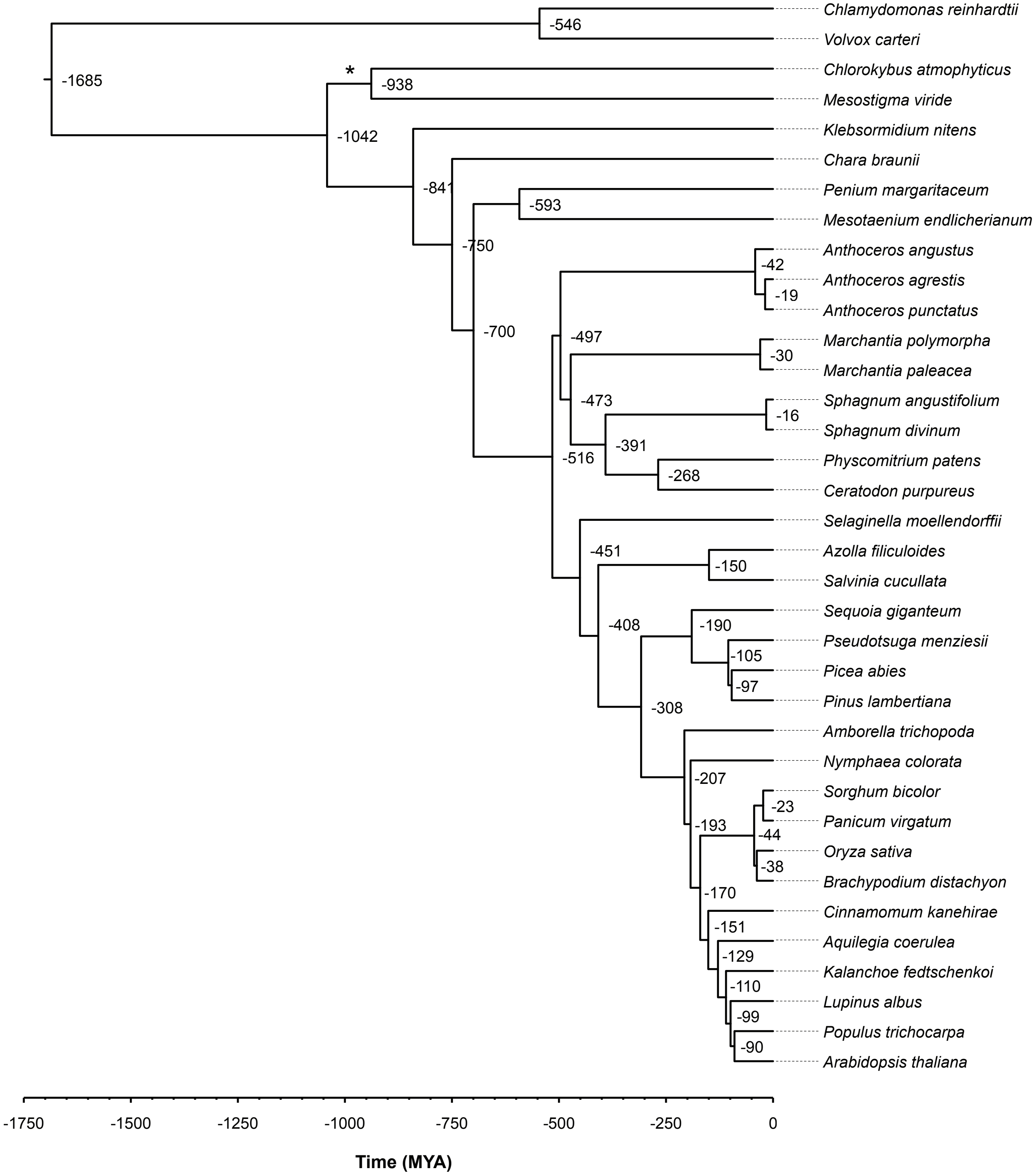

Supplemental Figure 2. Fossil calibrated land plant phylogeny with node ages [MYA = millions of years ago] labeled. All bipartitions received maximal support, except for the branch subtending the algae Chlorokybus and Mesostigma (*) which received an ultrafast bootstrap score of 98.

S. divinum wild population-MDS SNP variation per chromosome

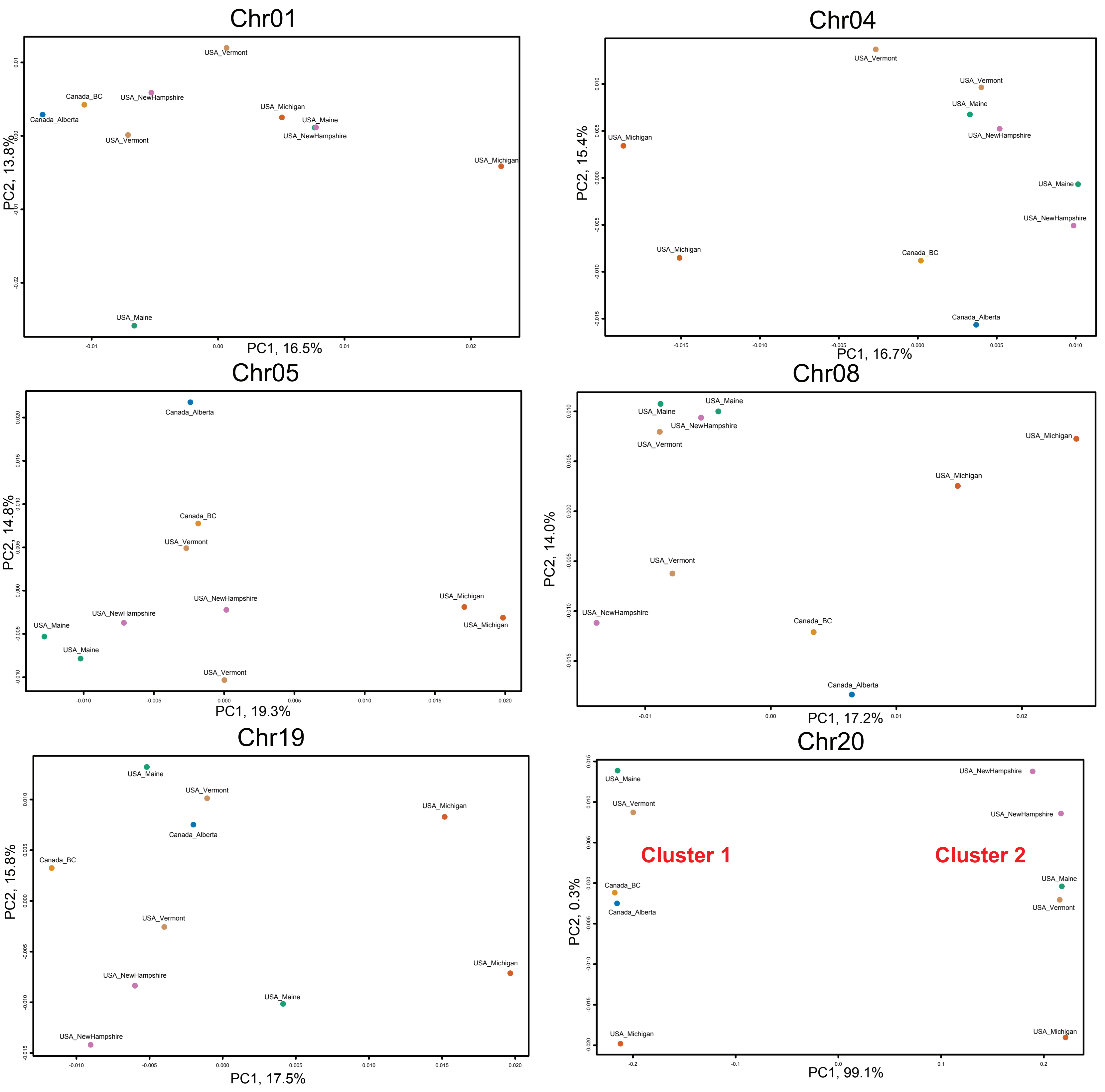

Supplemental Figure 3- SNP variation patterns per chromosome within North American populations of S. divinum. SNP variation per chromsome was extracted and used for multidimensional scaling (MDS) visualization. Points are colored based on shared location data, which is also provided as text. Two distinct clusters (labeled in red) were evident when visualizing variation present on chromosome 20.

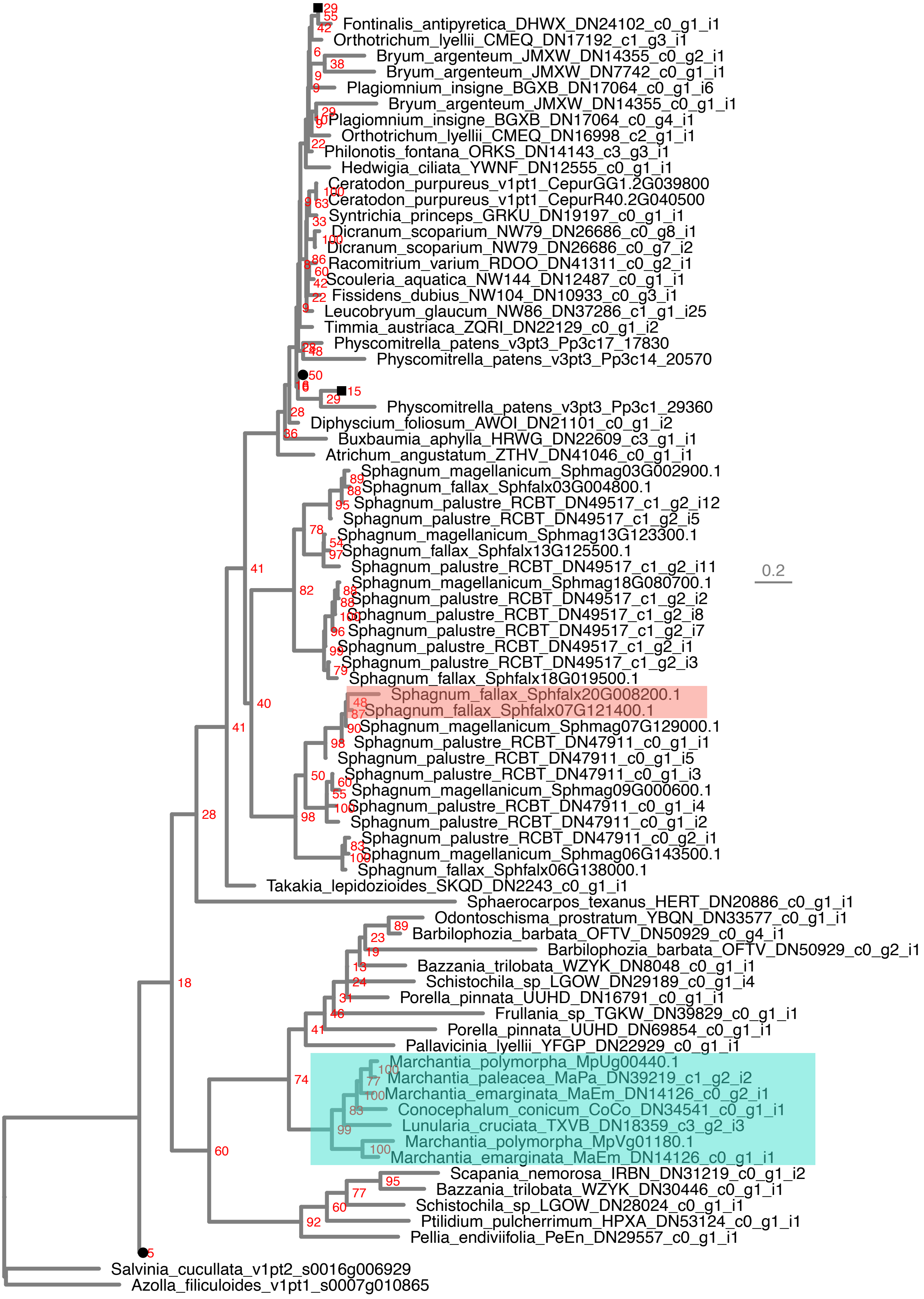

Supplemental Figure 4-Phylogenies of example sex-linked genes in Sphagnum and other bryophytes. Trees were built using Maximum-likelihood and figures were generated using ggtree v3.0.4 (Yu et al. 2017). Bootstrap support is shown in red, black circles represent collapsed clades, and the branch-length scale is in gray. The clades highlighted in salmon are sex-linked in Sphagnum and turquoise are sex-linked M. polymorpha. These topologies support independent captures onto the sex chromosomes across species, as the U and V-linked genes are more closely-related within a species than they are to other sex-linked copies.

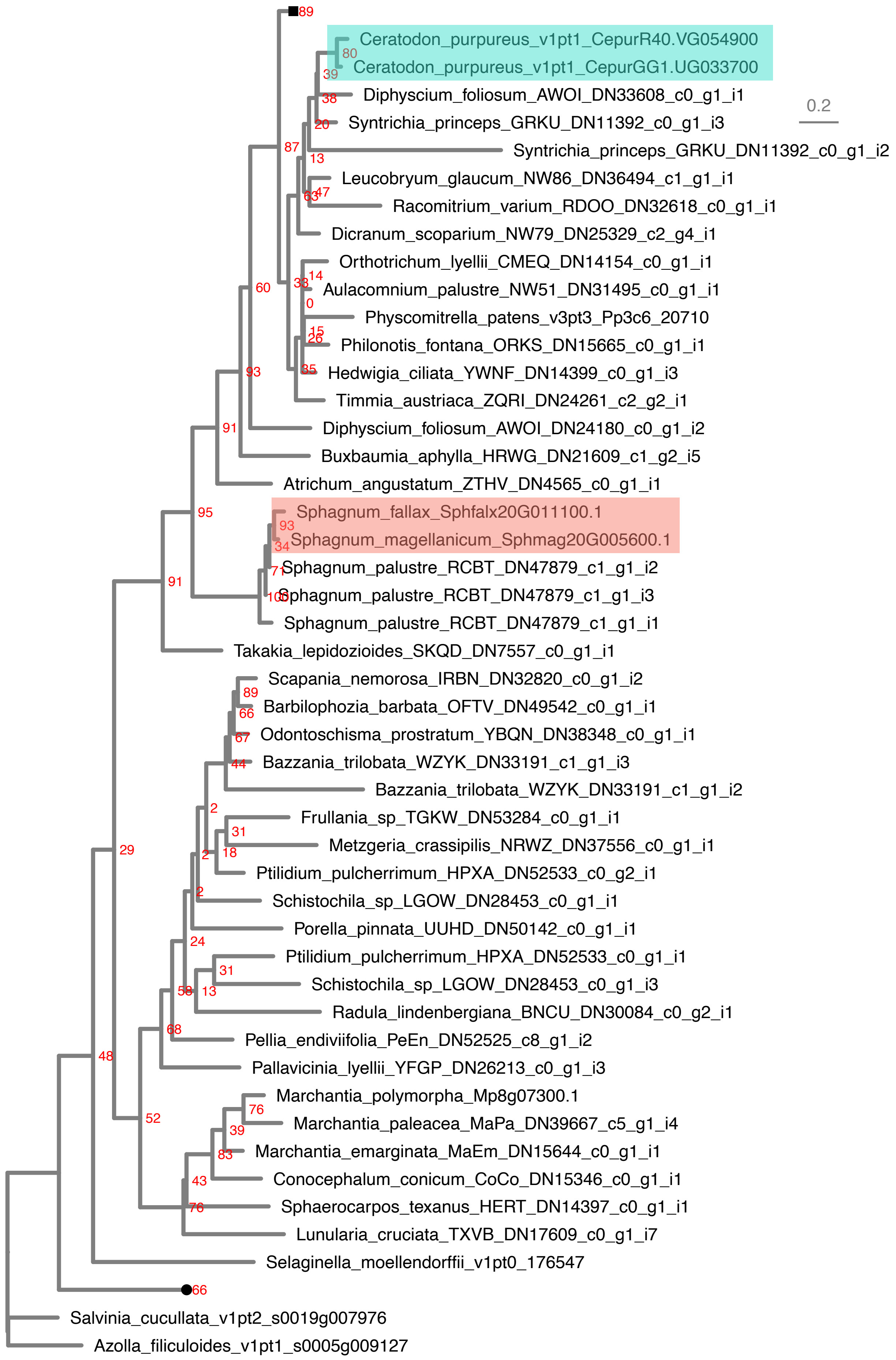

Supplemental Figure 5-Phylogenies of example sex-linked genes in Sphagnum and other bryophytes. Trees were built using Maximum-likelihood and figures were generated using ggtree v3.0.4 (Yu et al. 2017). Bootstrap support is shown in red, black circles represent collapsed clades, and the branch-length scale is in gray. The clades highlighted in salmon are sex-linked in Sphagnum and turquoise are sex-linked C purpureus. These topologies support independent captures onto the sex chromosomes across species, as the U and V-linked genes are more closely-related within a species than they are to other sex-linked copies.

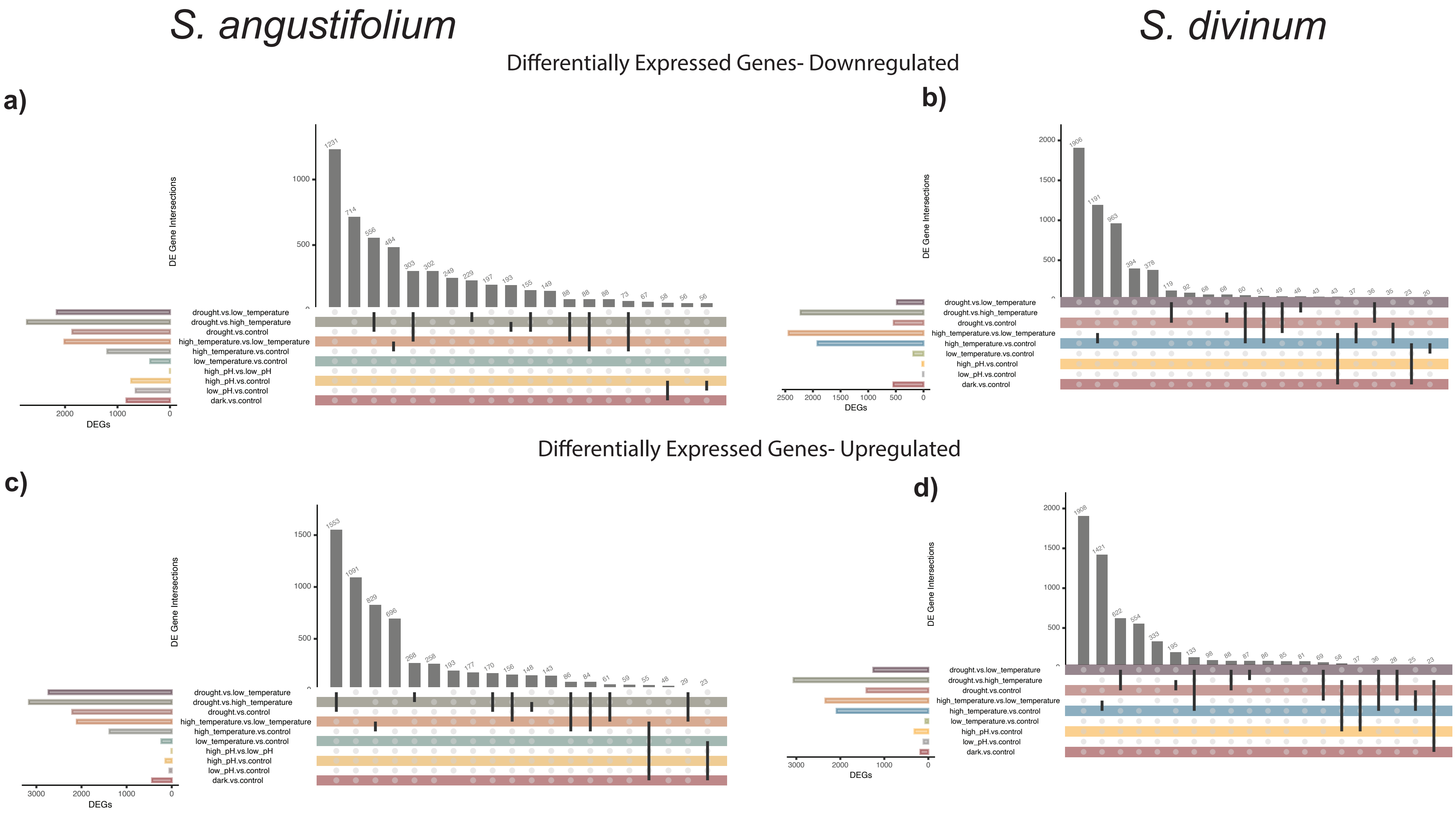

Supplemental Figure 6-Upset plots showing the number of overlapping differentially expressed genes detected among treatment comparisons in *Sphagnum* spp. a) downregulated genes in *S. angustifolium*, b) downregulated genes in *S. divinum*, c) upregulated genes in *S. angustifolium* and d) upregulated genes in *S. divinum*

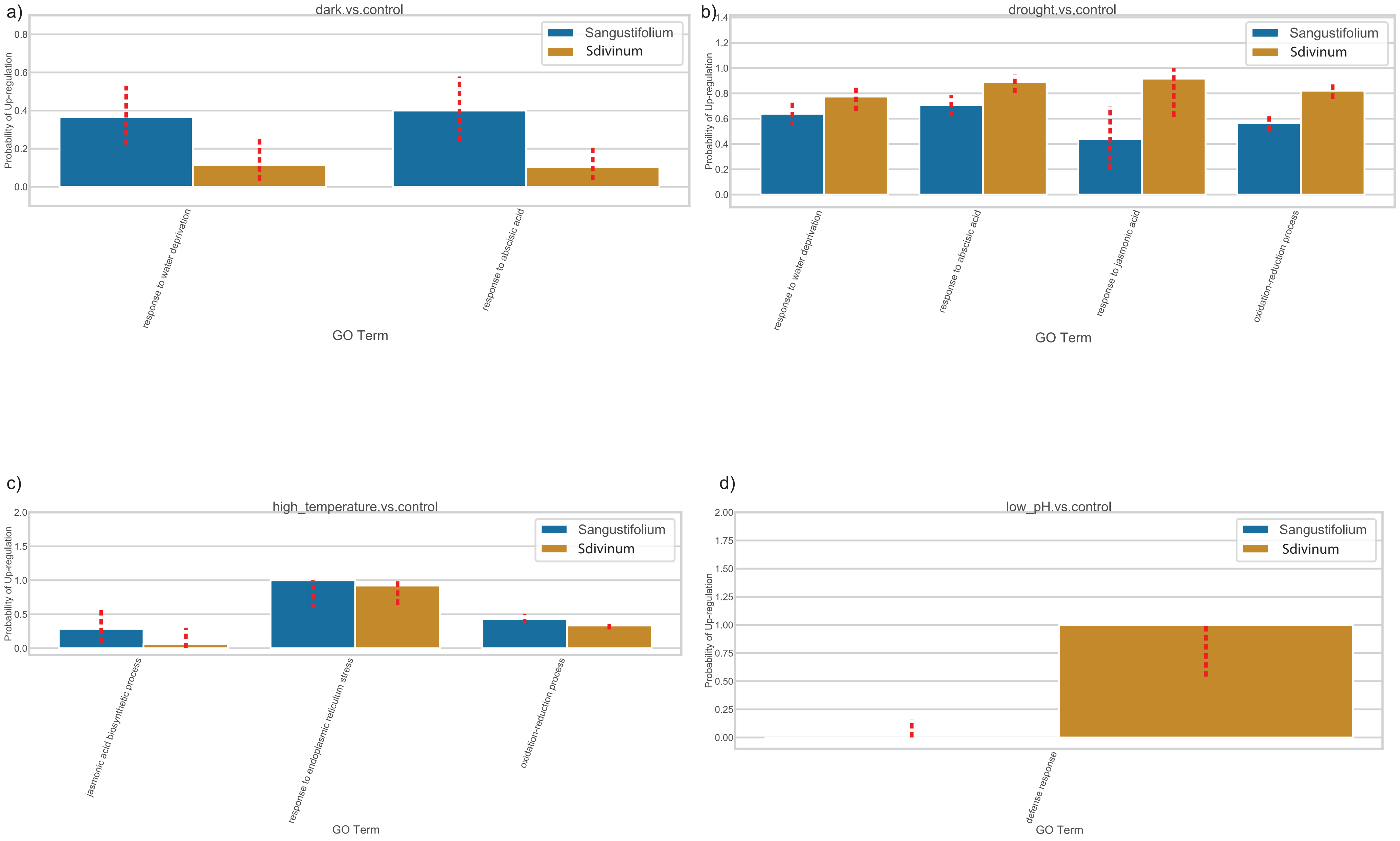

Supplemental Figure 7- Differentially expressed gene with shared Gene Ontology (GO) terms among *S. angustifolium* and *S. divinum*. A binomial test was used to determine the probability of genes with shared GO terms being up-regulated per condition per reference. Experimental Treatments: a) Dark treatment vs control (n = 149); b) drought vs control (n= 863); c) high temperature vs. control (n = 477); d) low pH vs control (n = 27).
Data are presented as values +/- SE (dotted line).

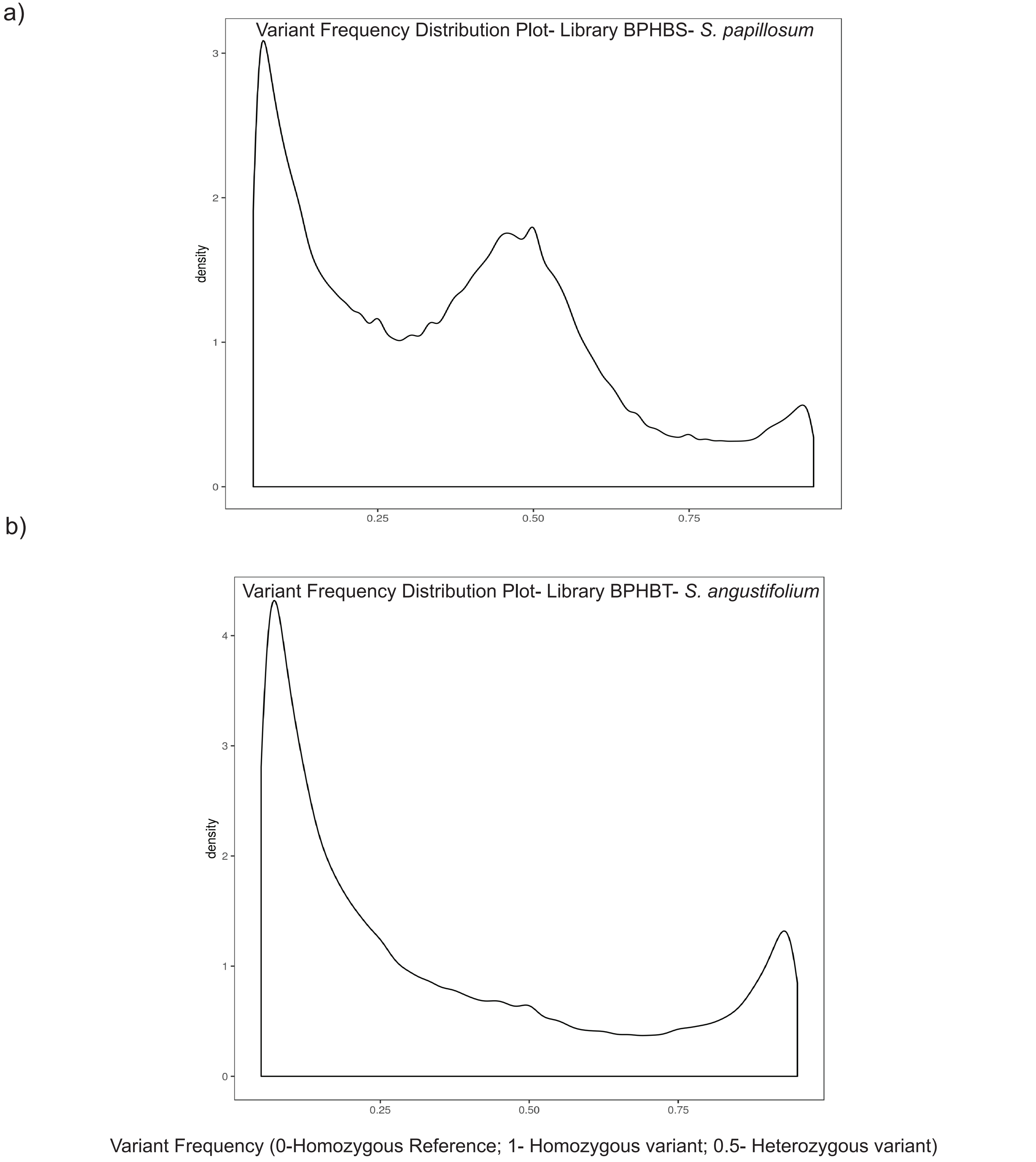

Supplemental Figure 8- Example variant frequency graphs to confirming polyploids within the *Sphagnum* diversity panel. Variant frequencies are calculated internally per sample. a) Variant frequency plot for library BPHBS- *S. papillosum*. b) Variant frequencly plot for library BPHBT- *S.angustifolium*. Haploid genotypes show only homozygous peaks (0 or 1), while diploid samples also display heterozygous variant peaks as well.
