# Supplementary material for: Newly identified sex chromosomes in the Sphagnum (peat moss) genome alter carbon sequestration and ecosystem dynamics
Source: Nat Plants. 2023 Feb 6;9(2):238–54. doi: 10.1038/s41477-022-01333-5 (PMC9946827; doi:10.1038/s41477-022-01333-5)
Supplement: Supplementary file 2 — Reporting Summary [file 41477_2022_1333_MOESM2_ESM.pdf]

nature portfolio

Corresponding author(s): Adam Healey

Last updated by author(s): Nov 23, 2022

Reporting Summary

Nature Portfolio wishes to improve the reproducibility of the work that we publish. This form provides structure for consistency and transparency in reporting. For further information on Nature Portfolio policies, see our Editorial Policies and the Editorial Policy Checklist.

Statistics

For all statistical analyses, confirm that the following items are present in the figure legend, table legend, main text, or Methods section.

n/a	Confirmed	
☐	☒	The exact sample size (*n*) for each experimental group/condition, given as a discrete number and unit of measurement
☐	☒	A statement on whether measurements were taken from distinct samples or whether the same sample was measured repeatedly
☐	☒	The statistical test(s) used AND whether they are one- or two-sided<br>*Only common tests should be described solely by name; describe more complex techniques in the Methods section.*
☒	☐	A description of all covariates tested
☒	☐	A description of any assumptions or corrections, such as tests of normality and adjustment for multiple comparisons
☐	☒	A full description of the statistical parameters including central tendency (e.g. means) or other basic estimates (e.g. regression coefficient) AND variation (e.g. standard deviation) or associated estimates of uncertainty (e.g. confidence intervals)
☐	☒	For null hypothesis testing, the test statistic (e.g. *F*, *t*, *r*) with confidence intervals, effect sizes, degrees of freedom and *P* value noted<br>*Give P values as exact values whenever suitable.*
☒	☐	For Bayesian analysis, information on the choice of priors and Markov chain Monte Carlo settings
☒	☐	For hierarchical and complex designs, identification of the appropriate level for tests and full reporting of outcomes
☒	☐	Estimates of effect sizes (e.g. Cohen's *d*, Pearson's *r*), indicating how they were calculated

Our web collection on statistics for biologists contains articles on many of the points above.

Software and code

Policy information about availability of computer code

Data collection	MECAT (v1.2); ARROW(v2.2.2); qtltools (v1.2.0); . Jellyfish (v2.3.0); Genomescope (v2.0); GSNAP (v2013-09-30);PASA (v2.0.2);EXONERATE(v2.4.0) ; RepeatMasker (v.open-4.0.7); RepeatModeler (v.open1.0.11); FGENESH+(v3.1.0); AUGUSTUS (v3.1.0); BRAKER1 (v1.6);BLASTP (2.2.26); HTSeq (v0.11.2); DESeq2 (v1.14.1); WGCNA R package (v1.49); topGO (v2.48.0); GOSemSim (v2.22.0);bwa-mem (v0.7.12);picard (v2.17.2-0); samtools (v1.9); Varscan (v2.4.3); bcftools (v1.9); plink (v1.9);GENESPACE (v0.9.4); MCScanX; Orthofinder (v2.5.4) ; MAFFT (v7.487); Mclust (v5.4.10); mixtools (v1.2.0); Broccoli (v1.2); DIAMOND (v0.9.35.136) ; FastTree (v2.1.11); translatorX (v1.1-2); trimAl (v1.2rev59); TreeShrink (v1.3.2); IQ-TREE2(v2.1.3) ; ModelFinder; treePL(v1.0); Count (v9.1106); AnnotationForge (v1.34.1); clusterProfiler (v4.0.5) ; GeMoMa (v1.6.4); ASTRAL (v5.7.1); NOVOPlasty (v2.6.7); phytools' (v0.7-90); BCFtools (v1.13); VCFtools (v0.1.17); STAR (v2.7.9a); GATK (v4.2.2.0); Dsuite (v0.4 r38);MACSE (v2.05);evobiR (v1.1);HyPhy (v2.5.32); PopGenome (v2.7.5) ;R version 4.0.3;karyoploteR (v1.16.0); BLAT (v30) ; bedtools (v2.29.0); RAxML (v8.2.12) ; R/qtl (v1.50)
Data analysis	All codes used for data analysis are open source (e.g. Python; R) and are freely available and deposited in Figshare.

For manuscripts utilizing custom algorithms or software that are central to the research but not yet described in published literature, software must be made available to editors and reviewers. We strongly encourage code deposition in a community repository (e.g. GitHub). See the Nature Portfolio guidelines for submitting code & software for further information.

Data

Policy information about availability of data

All manuscripts must include a data availability statement. This statement should provide the following information, where applicable:

- Accession codes, unique identifiers, or web links for publicly available datasets
- A description of any restrictions on data availability
- For clinical datasets or third party data, please ensure that the statement adheres to our policy

Additional work to support the findings of this manuscript can be found in the supplementary data section. Sequencing libraries (Illumina DNA/RNA and PacBio CLR) are publicly available within the sequence read archive (SRA). Individual accession numbers are provided in Supplemental Table 16, with additional data submitted under bioproject: PRJNA799298. Genome assemblies and annotations (v1.1) are freely available at Phytozome (https://phytozome-next.jgi.doe.gov/). These Whole Genome Shotgun projects have been deposited at DDBJ/ENA/GenBank under the accessions JAJQJK000000000 (S. angustifolium) JAKJHR000000000 (S. divinum). The versions described in this paper are versions JAJQJK010000000 and JAKJHR010000000. Raw data used for analysis in this manuscript are freely available on Figshare (https://doi.org/10.6084/m9.figshare.21232100).

Human research participants

Policy information about studies involving human research participants and Sex and Gender in Research.

Reporting on sex and gender	N/A
Population characteristics	N/A
Recruitment	N/A
Ethics oversight	N/A

Note that full information on the approval of the study protocol must also be provided in the manuscript.

Field-specific reporting

Please select the one below that is the best fit for your research. If you are not sure, read the appropriate sections before making your selection.

☒ Life sciences ☐ Behavioural & social sciences ☐ Ecological, evolutionary & environmental sciences

For a reference copy of the document with all sections, see nature.com/documents/nr-reporting-summary-flat.pdf

Life sciences study design

All studies must disclose on these points even when the disclosure is negative.

Sample size	No sample size calculations were performed. All available samples, provided sufficient data quality, were used for analysis.
Data exclusions	The Sphagnum resequencing libraries BPHAT, BPHAZ, BPHBZ, and BPHBS were excluded from phylogenetic analyses because contamination and/or low coverage prohibited de novo genome assembly.
Replication	Replication of PCR results were not attempted as results were consistent and independently obtained with each PCR reaction. For example, all male samples were 100 % positive for male specific primers and 100% negative for female specific primers in independent reactions. The same was true for all female samples (100% positive for female specific primers; 100% negative for male specific primers).
Randomization	Samples were not allocated into experimental groups for analysis.
Blinding	Samples were not allocated into experimental groups for analysis and thus, blinding was not necessary.

Reporting for specific materials, systems and methods

We require information from authors about some types of materials, experimental systems and methods used in many studies. Here, indicate whether each material, system or method listed is relevant to your study. If you are not sure if a list item applies to your research, read the appropriate section before selecting a response.

Materials & experimental systems

n/a	Involved in the study
☒	☐ Antibodies
☒	☐ Eukaryotic cell lines
☒	☐ Palaeontology and archaeology
☒	☐ Animals and other organisms
☒	☐ Clinical data
☒	☐ Dual use research of concern

Methods

n/a	Involved in the study
☒	☐ ChIP-seq
☒	☐ Flow cytometry
☒	☐ MRI-based neuroimaging
