# Supplementary figures and images for: Newly identified sex chromosomes in the Sphagnum (peat moss) genome alter carbon sequestration and ecosystem dynamics
Source: Nat Plants. 2023 Feb 6;9(2):238–54. doi: 10.1038/s41477-022-01333-5 (PMC9946827; doi:10.1038/s41477-022-01333-5)

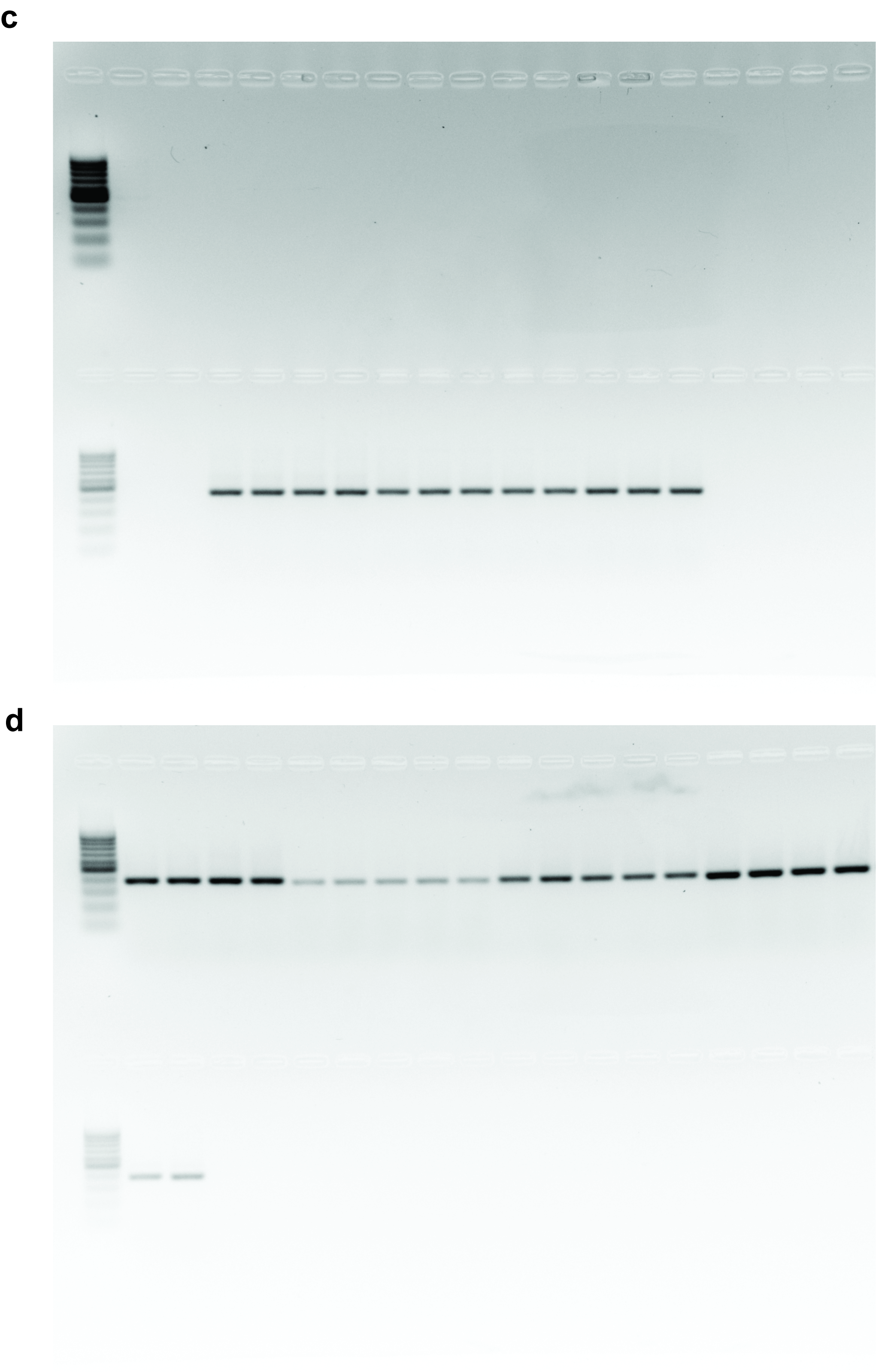

Supplement: Source Data Extended Data Fig. 3 — Unprocessed PCR gels for panels c and d. [file 41477_2022_1333_MOESM12_ESM.tif]
